# Early α-synuclein–mediated mitochondrial dysfunction in a human cell model of Parkinson’s disease dementia

**DOI:** 10.1038/s42003-026-10134-x

**Published:** 2026-05-12

**Authors:** Maha S. Alfaidi, Léa M. D. Wenger, Kornélia Szebényi, Thomas B. Stoker, Xiaoling He, Shaline V. Fazal, Jana Sebestikova, Amir Jassim, Richard J. Gilbertson, Raquel Garza, Johan Jakobsson, Maria Grazia Spillantini, Roger A. Barker, Wei-Li Kuan

**Affiliations:** 1 John van Geest Centre for Brain Repair and Department of Clinical Neuroscience, University of Cambridge, Cambridge, UK; 2 Department of Clinical Neurosciences, Cambridge University, Cambridge, UK; 3 Research Centre of Natural Sciences, Institute of Molecular Life Sciences, Budapest, Hungary; 4 Cancer Research UK Cambridge Institute, University of Cambridge, Li Ka Shing Centre, Robinson Way, Cambridge, UK; 5 Department of Oncology, University of Cambridge, The Early Cancer Institute, Cambridge, UK; 6 Aligning Science Across Parkinson’s (ASAP) Collaborative Research Network, Chevy Chase, MD USA; 7 Department of Experimental Medical Science, Wallenberg Neuroscience Centre and Lund Stem Cell Centre, BMC A11, Lund University, Lund, Sweden; 8 ALBORADA Drug Discovery Institute, Cambridge, UK; 9 Department of Neurology, Addenbrooke’s Hospital, Cambridge, UK; 10 Cambridge Stem Cell Centre, Cambridge, UK

**Keywords:** Cellular neuroscience, Parkinson's disease, Experimental models of disease, Neural ageing

## Abstract

Parkinson’s disease (PD) is a progressive neurodegenerative disorder characterised by the misfolding and accumulation of α-synuclein (α-syn) into pathological aggregates known as Lewy bodies. PD remains incurable, partly due to limited physiologically relevant models that recapitulate human pathology to enable therapeutic development. We developed a novel in vitro PD dementia model using fetal human cortical neurons seeded with α-syn preformed fibrils (PFFs). This model successfully replicates key PD features, including α-syn aggregation and mitochondrial gene dysregulation. Importantly, RNA sequencing revealed significant transcriptomic concordance between our model and PD postmortem tissue, particularly in the downregulation of mitochondrial genes linked to oxidative phosphorylation. We then evaluated two peptide inhibitors, β-syn36D (B36D) and S62. Both peptides demonstrated effective disaggregation of α-syn fibrils, with B36D showing particular promise by reversing PFF-induced functional and transcriptional changes to baseline levels. This human-relevant model captures essential pathological and transcriptomic disease hallmarks as well as demonstrating utility for therapeutic screening of drugs that block α-syn aggregation.

## Abstract (web summary)

A human neuron model of Parkinson’s disease using α-synuclein fibrils is developed, replicating key pathology and mitochondrial gene dysregulation, and tested peptide inhibitors B36D and S62.

## Introduction

Parkinson’s disease (PD) is the second most common neurodegenerative disorder after Alzheimer’s disease. It is characterised by the selective degeneration of specific neuronal populations in various brain regions, accompanied by insoluble cytosolic inclusions of α-synuclein (α-syn) called Lewy bodies (LB) and Lewy neurites^1^. It is a chronic and progressive condition, which manifests with a movement disorder consisting of tremor, rigidity, bradykinesia and postural instability, as well as non-motor features, including depression, sleep disturbances, autonomic dysfunction and anosmia. In addition, approximately 40% of patients develop dementia within 10 years of disease onset^2^, and this figure increases to 80% by 20 years^3^. The causal role of α-syn aggregation and LB formation in neurodegeneration is not fully understood. Point mutations in *SNCA* (A53T)^4^, the gene that encodes α-syn, is known to cause rare forms of autosomal dominant PD and dementia with Lewy bodies (DLB—another neurodegenerative α-synucleinopathy), supporting a central role for α-syn in the pathogenesis of these conditions. Several other point mutations (A53E, G51D, H50Q, E46K, and A30P) have been associated with the development of familial forms of PD and DLB^5,6^ In addition to these missense mutations, a recent study reported a novel *SNCA* mutation involving a 21-base pair duplication that leads to the insertion of seven amino acids (MAAAEKT) after residue 22 of α-syn, resulting in a protein of 147 amino acids in a case of juvenile-onset synucleinopathy^7^. Subsequent to the identification of SNCA gene mutations in these familial cases, α-syn has been found to be a major component of LB in both sporadic and familial PD^8^, with pathological α-syn heavily phosphorylated at serine 129, a modification that is the dominant disease-associated form found in LB^9^, directly linking α-syn to common sporadic forms of PD.

In addition to its role in forming insoluble aggregates and LBs, the oligomeric forms of α-syn are postulated to drive the underlying neurotoxicity associated with PD, and to serve as templates to seed further aggregation^10^. Disease-relevant neuronal phenotypes have been demonstrated by us and others, both in vitro and in vivo, through the administration of α-syn preformed fibrils (PFFs)^11–14^, offering a platform for understanding the toxic role of α-syn in these conditions. In this study, we utilised human fetal neurons to characterise the transcriptomic and phenotypic changes induced by PFF seeding and α-syn aggregation and compared these to those found in human PD post-mortem brain tissue. Additionally, we investigated the potential of two peptide inhibitors, B36D and S62, to modulate α-syn pathology and, consequently, disease progression.

## Results

### Characterisation of α-syn PFF uptake and α-syn pathology in fetal human cortical neurons

**Fig. 1:**
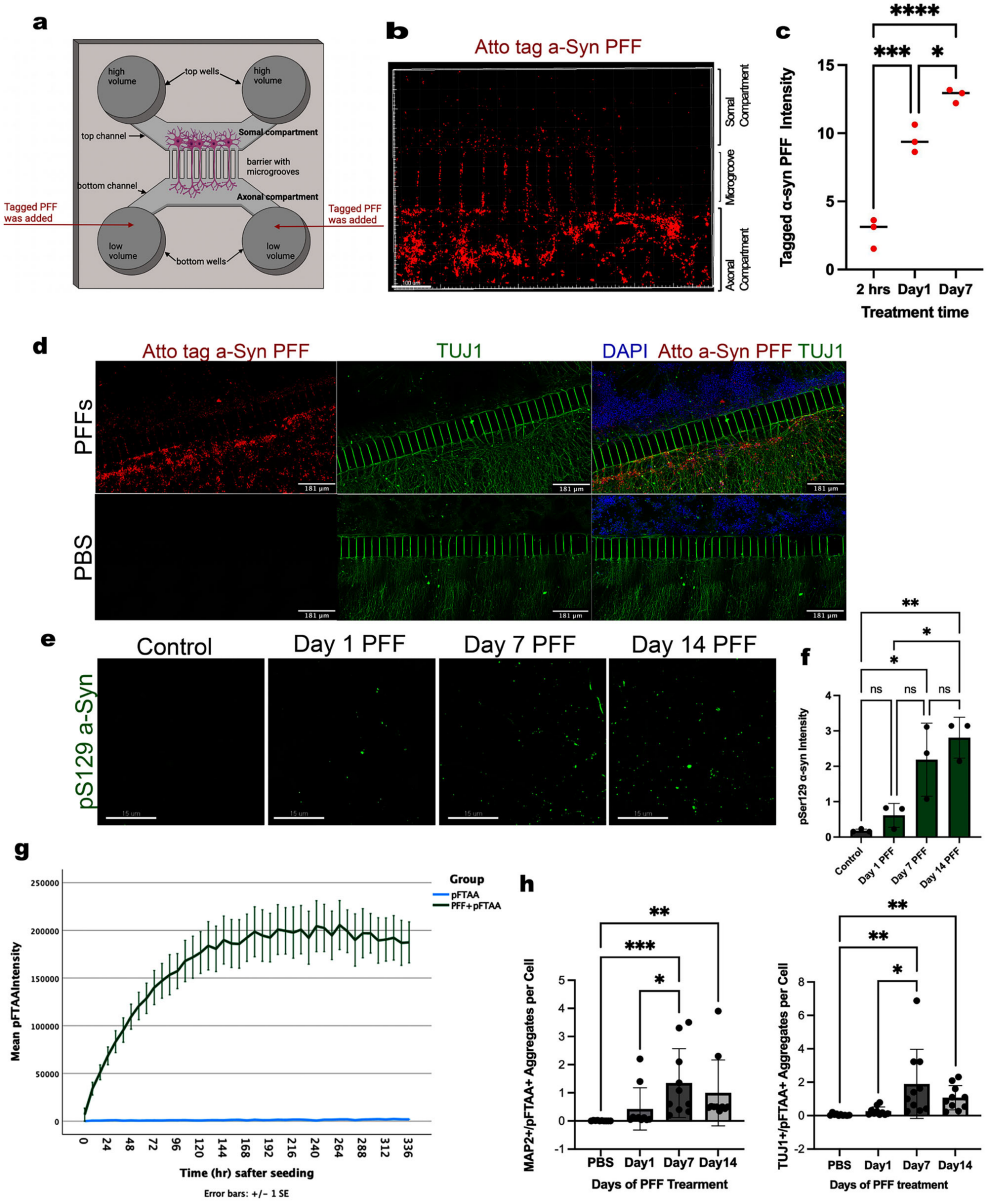
Characterisation of α-synuclein PFF and fetal human cortical model. **a** Schematic representation of human cortical neuronal plating protocol and selective seeding of tagged α-syn PFFs in the bottom chamber at 14 *DIV*. **b** Higher magnification confocal image (×40) of ATTO 594-tagged α-syn PFF immunostaining at 7 days post-treatment, showing positive signals in the axonal compartment (bottom chamber), microgrooves, and the somal compartment (top chamber). Scale bars = 100 µm. **c** Quantification of tagged α-syn PFF signal intensity in microgrooves, and top chamber across treatment durations (2 h, 1 day, and 7 days), showing a significant increase over time. One-way ANOVA with Tukey’s multiple comparison test: *F*(2.8, 9.5) = 12.9, *p* = 0.0002; *F*(2.8, 12.8) = 19.1, *p* < 0.0001; *F*(9.5, 12.8) = 6.2, *p* = 0.011, *n* = 3 biological replicates. **d** Representative lower magnification confocal images (×20) of control and PFF-treated samples. Red: tagged α-syn PFF; green: neuronal marker TUJ1; blue: DAPI nuclear stain. Scale bars = 181 µm. **e** Confocal images showing pS129 α-syn-positive staining at 1, 7, and 14 days post-PFF treatment. Scale bars = 15 µm. **f** Quantification of pS129 α-syn fluorescence intensity across treatment durations. One-way ANOVA with Tukey’s multiple comparison test: *F*(0.18, 2.18) = 5.65, *p* = 0.01; *F*(0.18, 2.81) = 7.41, *p* = 0.003; *F*(0.61, 2.81) = 6.2, *p* = 0.01, *n* = 3 independent biological replicates; error bars represent mean ± SD. **g** Live imaging analysis showing pFTAA α-syn PFF intensity over 14 days, demonstrating aggregation kinetics, *n* = 5 independent replicates; error bars represent mean ± SE. **h** Colocalisation analysis of pFTAA-positive PFF signal with neuronal markers MAP2 and TUJ1, showing a significant increase at 7 days, and 14 days PFF post-treatment. The mean intracellular spot count of pFTAA+ aggregates co-localised with the neuronal marker was normalised to DAPI to account for cell number. One-way ANOVA with Tukey’s multiple comparison test: MAP2/pFTAA colocalisation: *F*(0.006, 1.35) = 6.97, *p* = 0.0002; *F*(0.006, 0.99) = 5.2, *p* = 0.005; *F*(0.43, 1.35) = 4.8, *p* = 0.01. TUJ1/pFTAA colocalisation: *F*(0.05, 1.7) = 5.4, *p* = 0.006; *F*(0.05, 1.89) = 6.4, *p* = 0.003; *F*(0.26, 1.89) = 4.83, *p* = 0.01, *n* = 5 biological replicates with duplicates per condition; error bars represent mean ± SD.

We used a microfluidic chamber to assess the intracellular uptake of α-syn PFFs in human fetal cortical neurons through the terminal axonal compartment (Fig. 1a). Using the microfluidic system, we measured PFF uptake under defined flow and pressure gradients, which allowed us to quantify cellular internalisation under controlled conditions. Our findings align with previous studies reporting that PFFs are internalised by cells through regulated uptake pathways^12^. We found that seeding with Atto 594-tagged α-syn PFF in the terminal axonal compartment resulted in efficient uptake and retrograde transport to neuronal cell bodies within 24 h (Fig. 1b–d). PFF treatment also resulted in hyperphosphorylation of α-syn at serine 129 (pS129) at multiple time points (1, 7, and 14 days post-seeding), providing further evidence that seeding induces relevant pathological changes in endogenous α-syn (Fig. 1e, f). We acknowledge the possibility that some of the detected pS129 signal is attributable to the pS129 present in the PFF seeds^15^. However, our interpretation that the observed signal represents pathologically relevant, de novo phosphorylation of endogenous α-syn is based on the following quantitative and morphological evidence. Firstly, the increase in the pS129 signal detected in the PFF-treated neurons is quantitatively far greater than the basal level of pS129 introduced with the PFF seeds (Fig. 1e, f). Furthermore, this pS129 signal accumulates over the culture period (e.g., several days to weeks post-seeding), a time-dependent increase consistent with PFFs activating host cell kinases (like CK1/2 or G-protein receptor kinases) to hyperphosphorylate native α-syn. Secondly, the observed pS129 staining co-localises with the formation of widespread intracellular inclusions within the cell cytoplasm and neurites (Fig. 1e, f). This morphological hallmark is the defining feature of seeding-induced pathology and is a product of the cell’s response to the aggregate, not a simple measurement of the initial seed material.

To monitor PFF seeding dynamics in real-time, we used pentameric formyl thiophene acetic acid (pFTAA), a cell-permeable, conformation-selective dye previously validated in our animal models^13^. Live imaging over a 14-day period revealed dynamic changes in protein aggregation, characterised by a progressive increase in pFTAA signal intensity during the first 4–5 days, followed by a plateau phase (Fig. 1g). Then, we examined the cell-type specificity of PFF-induced pathology and found it in (MAP2 and TUJ1) neurons (Fig. 1h). In addition, pFTAA signals were also detected in GFAP-positive cells, indicating that PFF-induced pathology extends to astrocytes, which can also internalise and process α-syn PFFs (Supplementary Fig. 1a, b). pFTAA is widely used as a conformation-sensitive dye for amyloid-like aggregates, including α-syn, in various cell types such as neurons and microglia^16^. To ensure specificity in our system, we performed co-labelling with GFAP (far-red channel, 740 nm) to mark astrocytes, while pFTAA signal was detected in the green channel. The pFTAA signal exhibited distinct punctate morphologies, consistent with neuronal α-syn inclusions rather than filamentous astrocytic structures. These observations confirm that pFTAA primarily detects α-syn aggregates in neurons rather than GFAP filaments in astrocytes. Since the focus of our study is on neuronal α-syn pathology, the relevant astrocyte data are included only in the Supplementary Information for reference.

### scRNA-seq reveals downregulation of mitochondrial genes in the PFF fetal human in vitro model

Next, we sought to determine whether the PFF application resulted in relevant downstream pathology in human fetal cortical neurons. We performed 10X single-cell RNA sequencing (scRNA-seq) on cells treated with α-syn PFFs for 14 days. Human fetal cortical neurons were cultured under control and PFF-treated conditions, after which approximately 20,000 live cells per sample were collected for scRNA-seq analysis. To ensure sample viability, 3 million live cells from each condition were tested using flow cytometry (FACS), revealing high viability (>93%) across all samples (Supplementary Fig. 2a).

**Fig. 2:**
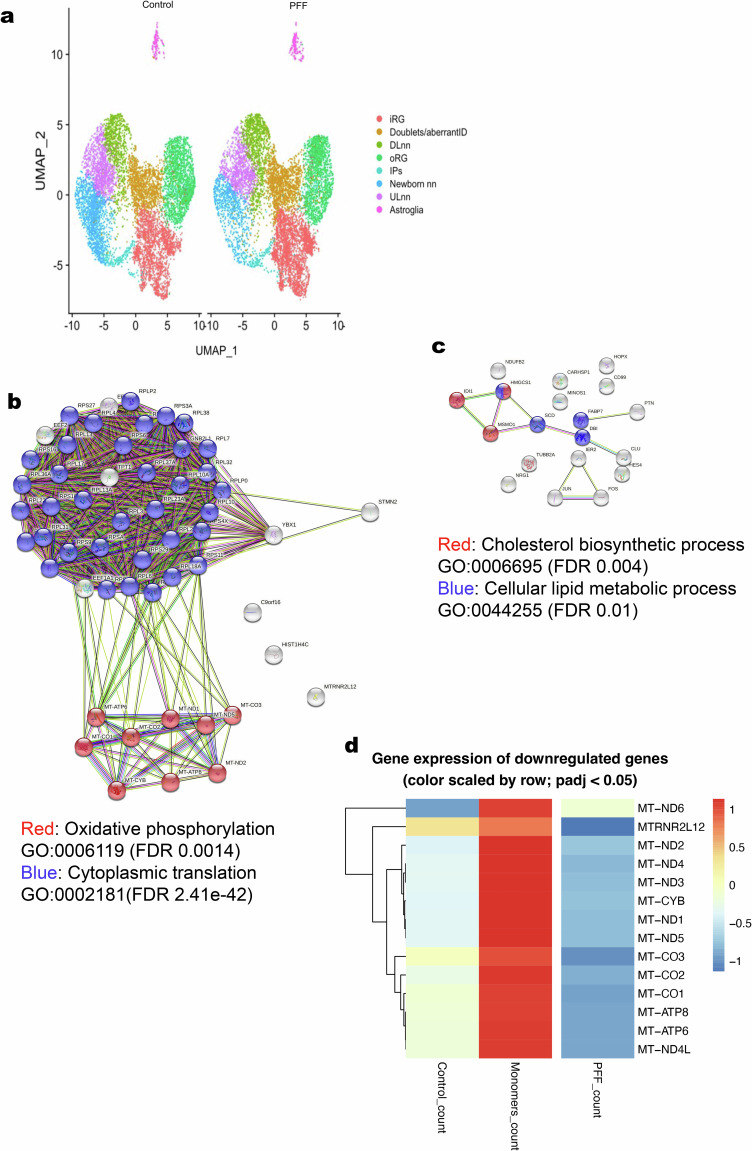
Fetal human cell types and transcriptional profile reveal mitochondrial pathway dysregulation and cholesterol/lipid metabolic process alterations. **a** Cell types in fetal cortical neurons under control (untreated) and PFF-treated (pathological) conditions, clustered by cell type (colour-coded). Each dot represents a single cell. Cell types include inner radial glia (iRG, red), outer radial glia (oRG, dark green), deep-layer neurons (DLnn, light green), upper-layer neurons (ULnn, purple), newborn neurons (Newborn nn, blue), intermediate progenitors (IPs, tiffany blue), astroglia (pink), and doublets (yellow). **b** Downregulated genes in the PFF-treated group compared to controls. Red indicates mitochondrial genes associated with oxidative phosphorylation (GO:0006119, FDR 0.0014), while blue represents ribosomal genes linked to the cytoplasmic translation pathway (GO:0002181, FDR 2,41e-42). **c** Upregulated genes in the PFF-treated group compared to controls. Red indicates genes involved in cholesterol biosynthesis (GO:0006695, FDR 0.004), and blue represents genes associated with cellular lipid metabolic processes (GO:0044255, FDR 0.01). **d** Heatmap showing the expression of the same downregulated genes identified in the scRNA-seq dataset (as in **b**, Red). Bulk RNA-seq analysis confirms that these mitochondrial genes (*MT-ATP6, MT-ATP8, MT-ND1, MT-ND2, MT-ND5, MT-CO1, MT-CO2, MT-CO3*, and *MT-CYB*) are downregulated specifically under the α-syn PFF condition, but not with monomeric α-syn treatment. Blue represents decreased gene expression, indicating that the transcriptional effects are driven by α-syn PFFs rather than α-syn monomers.

Using scRNA-seq, we characterised the transcriptomic profiles of fetal human cortical neurons following PFF treatment. Multiple mature neuronal subpopulations were identified, including deep-layer neurons (DLn) marked by *CTP2* and *FEZF2* and upper-layer neurons (ULn) marked by *SATB2* and *CUX2*, both of which represent excitatory neuronal types. Additionally, newborn neurons, expressing early differentiation markers *SOX11* and *NEUROD2*, were detected alongside astroglia identified by *GFAP* and *S100B*. Progenitor subtypes included inner radial glia (iRG) expressing *TOP2A*, outer radial glia (oRG) expressing *HOPX*, and intermediate progenitors (IPs) expressing *EOMES* (Fig. 2a).

Comparative analyses of control and PFF-treated cells revealed a similar cellular cluster profile using UMAP; however, a reduction in the number of newborn neurons and an enrichment of astroglia was observed in the PFF condition (Fig. 2a). This finding suggests that PFF exposure selectively affects the vulnerability of developing neurons, and enhanced astroglial proliferation may act as a reactive response. The selective vulnerability of newborn neurons and the proliferative response of astroglia further highlight the diverse cellular effects of PFF exposure.

Differential gene expression (DGE) analysis revealed significant transcriptional changes in response to PFF treatment. Downregulation of mitochondrial and ribosomal genes was prominent across ULn, newborn neurons, and oRG populations. Specifically, genes involved in oxidative phosphorylation and ATP synthesis, including *MT-ATP6, MT-ATP8, MT-ND1, MT-ND2, MT-ND5, MT-CO1, MT-CO2, MT-CO3*, and *MT-CYB*, were significantly downregulated (GO:0006119, FDR = 0.0014) (Fig. 2b). This coordinated downregulation suggests possible mitochondrial dysfunction and impaired energy metabolism. These findings suggest that mitochondrial impairment may represent a potentially early event in response to PFF treatment. In addition to mitochondrial deficits, PFF treatment induced significant upregulation of genes (*SCD, IDI1, MSMO1, DBI, HMGCS1*, and *FABP7*) involved in cholesterol biosynthesis (GO:0006695, FDR = 0.004) and lipid metabolism (GO:0044255, FDR = 0.01) within the oRG population (Fig. 2c). The observed alterations in cholesterol biosynthesis and lipid metabolism pathways following PFF treatment may contribute directly to mitochondrial dysfunction, rather than solely reflect a downstream response to aggregate-induced stress. This possibility is supported by prior studies demonstrating that lipid accumulation and disruption of membrane homoeostasis can induce mitochondrial toxicity and impair respiratory function^17^. Thus, dysregulated lipid metabolism may represent a mechanistic link between α-syn aggregation and mitochondrial impairment in our model.

To clarify whether monomeric α-syn contributes to the residual transcriptional dysregulation observed after peptide treatment, we performed an additional bulk RNA-sequencing experiment using an independent batch of primary human fetal cortical neurons. This analysis directly compared the transcriptional profiles of neurons treated with α-syn PFFs vs. monomeric α-syn. Neurons exposed to PFFs showed a marked downregulation of mitochondrial-associated genes (*MT-ATP6, MT-ATP8, MT-ND1, MT-ND2, MT-ND5, MT-CO1, MT-CO2, MT-CO3*, and *MT-CYB*) (Fig. 2d), consistent with the pathology identified in our scRNA-seq dataset (Fig. 2b). In contrast, monomeric α-syn did not induce comparable transcriptomic alterations, indicating that this species alone is insufficient to drive the disease-related transcriptional phenotype. These findings support the consensus that fibrillar or oligomeric α-syn species are the toxic form of this protein and confirm that the monomeric products generated following B36D/S62-mediated disaggregation do not underlie the residual dysregulation. Instead, we attribute the incomplete rescue either to uncleared oligomeric/fibrillar species that persist despite treatment or to downstream cellular damage that may be irreversible. Together, these data strengthen the conclusion that B36D/S62 peptides effectively reduce the pathological aggregate burden while improving neuronal function and transcriptional homoeostasis.

### Transcriptional changes in fetal human PFF model compared to PD post-mortem brain material

**Fig. 3:**
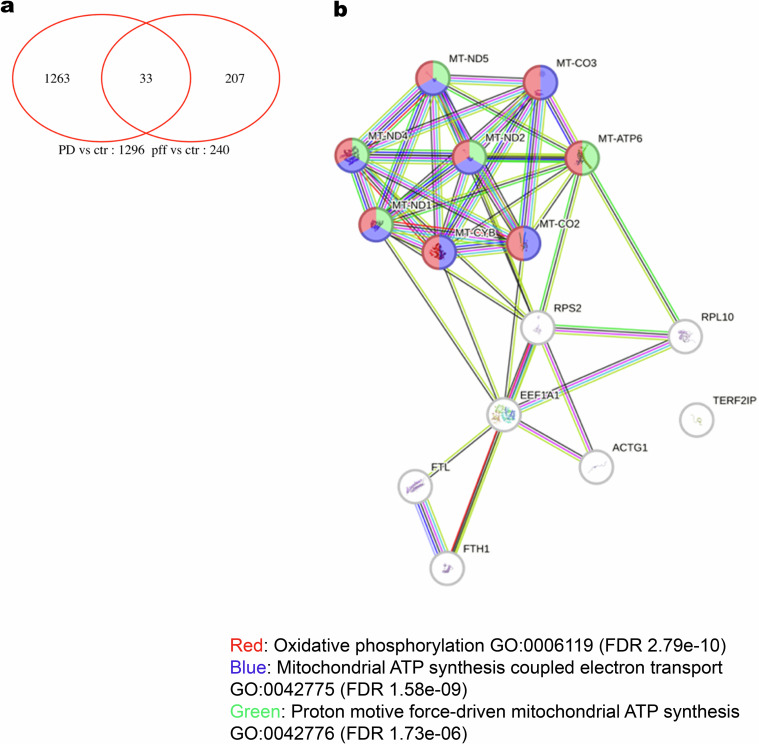
The fetal PFF model develops mitochondrial dysregulation similar to that in postmortem Parkinson’s disease (PD) material. **a** Differential gene expression analysis reveals an overlap between postmortem PD samples and the PFF-treated fetal model. A total of 33 genes were similarly altered in the PD postmortem tissue and PFF-treated fetal neurons compared to their respective controls. **b** Both the PFF model and PD postmortem material show downregulation of the same mitochondrial genes associated with oxidative phosphorylation (red:GO:0006119, FDR 2.79e−10), ATP synthesis (blue: GO:0042775, FDR 1.58e−09, green:GO:0042776, FDR 1.73e−06), and other key mitochondrial pathways, indicating shared mitochondrial dysfunction.

To validate the biological relevance of our fetal human PFF model, we next compared scRNA-seq detected transcriptional changes induced in fetal cortical neurons by PFFs to those seen in the prefrontal cortex of post-mortem brains from idiopathic PD patients, detected by single nucleus RNA-seq (snRNA-seq) data from post-mortem PD and control brain tissues. We analysed the differentially expressed genes in both datasets by comparing the PFF-treated fetal human cortical neurons to control fetal neurons (Fig. 3a), and PD post-mortem brain tissue to control post-mortem brain tissue. Using gene set enrichment analysis (GSEA), we identified 33 common differentially expressed genes from the two datasets (Fig. 3a). The most compelling finding was the consistent downregulation of mitochondrial genes observed in both the fetal PFF-treated samples and the PD postmortem brain datasets, specifically those involved in oxidative phosphorylation. Key affected genes included components of the electron transport chain: ATP synthase (*MT-ATP6*), NADH dehydrogenase subunits (*MT-ND1, MT-ND2, MT-ND4, MT-ND5*), cytochrome b (*MT-CYB*), and cytochrome c oxidase subunits (*MT-CO2, MT-CO3*) (Fig. 3b). This shared pattern of mitochondrial dysfunction suggests that impaired energy metabolism may be the earliest feature of an α-synucleinopathy. Despite the differences in age and pathology between the fetal PFF model and the PD post-mortem brain samples, there was a significant downregulation of mitochondrial genes in both datasets (Fig. 3b). These findings suggest that our fetal human PFF model is a valuable tool for studying PD/PDD pathogenesis.

### Peptide inhibitors reverse PFF-induced α-syn aggregation

**Fig. 4:**
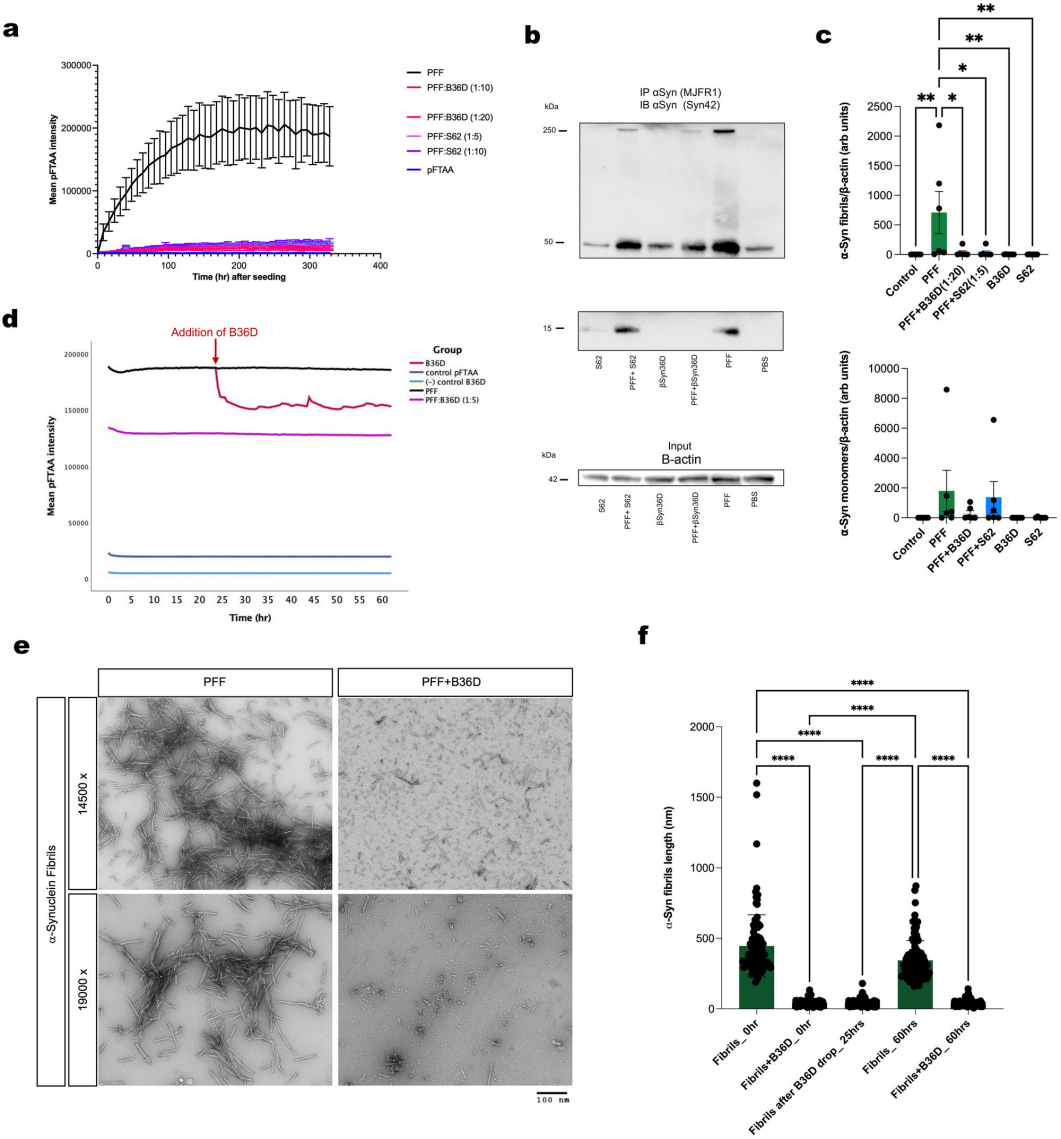
Peptide inhibitors disaggregate α-synuclein preformed fibrils and fibrils in vitro and in cell-free culture. **a** Live imaging over 14 days using pFTAA fluorescence intensity under various conditions: PFF (70 nM) (positive control, black), PFF: B36D (1:10, light pink), PFF: B36D (1:20, dark pink), PFF: S62 (1:5, light purple), PFF: S62 (1:10, dark purple), and pFTAA alone (negative control, dark blue). Fetal cortical neurons (7 DIV) were treated with these combinations of agents, and images were captured every 8 h. **b** Immunoprecipitation analysis of α-syn species in treated fetal cortical neurons. Conditions: PBS, PFF alone, PFF: B36D (1:20), B36D alone, PFF: S62 (1:5), and S62 alone. Upper blot shows HMW α-syn species; lower blot shows monomeric α-syn. PFF-alone samples showed elevated HMW species, while peptide inhibitor treatments reduced HMW levels. Both PFF conditions maintained prominent monomer bands. **c** Quantitative assessment of immunoprecipitated α-syn HMW and monomer levels across treatment conditions. One-way ANOVA with Dunnett’s multiple comparisons test: **p = 0.007, *F*(710.2, 1) = 3.4; *p = 0.1, *F*(710.2, 33) = 3.2; *n* = 6 biological replicates. **d** Cell-free kinetic assay of pFTAA fluorescence measured using a plate reader across different conditions: α-syn fibrils (unsonicated PFF; 35 µM) (black), α-syn fibrils:B36D at a 1:5 ratio (35 µM:175 µM) (pink), α-syn fibrils with an additional dropwise addition of B36D (35 µM) (red), and negative controls including B36D alone (35 µM) (light blue) and pFTAA alone (2 µM) (dark blue). **e** TEM analysis at multiple timepoints (0 h, post-B36D addition, and 60 h), comparing untreated and B36D-treated fibril morphology. **f** Quantitative analysis of TEM data comparing fibril length across timepoints and treatment conditions. Statistical comparisons using one-way ANOVA with Tukey’s multiple comparisons test: Fibrils_0 h vs. Fibrils + B36D_0 h: *F*(446.2,40.78) = 36.09, *p* < 0.0001, Fibrils_0 h vs. Fibrils after B36D drop_25 h: *F*(446.2,43.80) = 35.82, *p* < 0.0001, Fibrils_0 h vs. Fibrils + B36D_60 h: *F*(446.2,42.67) = 35.92, *p* < 0.0001, Fibrils + B36D_0 h vs. Fibrils_60 h: *F*(40.78,344.1) = 27.00, *p* < 0.0001, Fibrils after B36D drop_25 h vs. Fibrils_60 h: *F*(43.80,344.1) = 26.73, *p* < 0.0001, Fibrils_60 h vs. Fibrils + B36D_60 h: *F*(344.1,42.67) = 26.83, *p* < 0.0001,*n* = 3 replicates. Experiments in **a**–**c** were conducted in fetal cortical human cell cultures, while **d**–**f** represent cell-free conditions. Error bars represent mean ± SD from independent replicates unless otherwise stated.

Clearance of protein aggregates (amyloid-β) has been shown to confer clinical benefit in Alzheimer’s disease—another neurodegenerative proteinopathy^18^. We therefore sought to investigate whether the clearance of misfolded α-syn could reverse the downstream pathology in our PFF-seeded human fetal neurons, by treatment with the peptide inhibitors B36D and S62, both previously shown to be active in cells^19,20^, These peptides are designed to bind specifically to α-syn, with B36D targeting the NAC region to prevent aggregation and S62 binding to the C-terminal region to promote disaggregation of existing fibrils. We first analysed the dose-dependent effect of peptide inhibitors on protein aggregation by measuring pFTAA kinetics with live cell imaging; peptide inhibitors were added at the same time as PFF seeding in ratios of PFF: B36D (1:10), (1:20) and PFF: S62 (1:5), and (1:10). Peptide inhibitors significantly reduced protein aggregation under all conditions, compared to control (Fig. 4a). We then tested the ability of these peptide inhibitors to lower the high molecular weight (HMW) forms of α-syn. Using immunoprecipitation with the C-terminus α-syn antibody (MJFR1), we demonstrated a significant reduction of HMW α-syn after 14 days of treatment with both peptide inhibitors (Fig. 4b, c). The signal observed at 15 kDa (of monomeric α-syn) is likely caused by the breakdown of PFFs during sample preparation, as opposed to endogenous α-syn (Fig. 4b). The monomer-sized band migrating at approximately 15 kDa in the PFF and PFF + S62 groups (Fig. 4b) can be a technical artefact resulting from the denaturation and heating of residual α-syn aggregates during sample preparation, as previously described^21^. The presence of this band directly correlates with the amount of uncleared PFF material available for disassembly in those samples, rather than representing differences in the physiological abundance of endogenous monomeric α-syn.

In the input lanes of the α-syn immunoprecipitation experiments (Fig. 4b and Supplementary Fig. 3d), a protein band at 42 kDa corresponds to β-actin, which was used as a loading control. All experimental conditions were stained with β-actin to confirm equal protein loading, ensuring that observed differences in α-syn immunoprecipitation are not attributable to variations in sample protein content. This control validates the quantitative interpretation of monomeric or fibrillar α-syn detected across treatment groups.

The disaggregation potential of these peptides was further investigated using a cell-free kinetic assay. We then assessed whether peptide inhibitors could disaggregate existing α-syn fibrils. Using the cell-free kinetic assay, we recorded a rapid reduction of pFTAA signal upon the addition of B36D (35 µM) to unsonicated PFF (35 µM) (Fig. 4d). To understand the structural phenotypic changes and find an explanation for the drop in pFTAA signal observed post-inhibitor treatment, we examined samples collected from the kinetic cell-free experiment using transmission electron microscopy (TEM). This allowed us to evaluate the ultrastructural features of unsonicated PFF with and without B36D treatment. We found a significant reduction in the length and number of α-syn fibrils in samples treated with B36D (Fig. 4e, f) and S62 (Supplementary Fig. 3e, f). The disaggregation effect of B36D treatment was also observed in live cell imaging (Supplementary Fig. 3c) in the absence of a microglial population. These results highlight the therapeutic potential of molecules acting through mechanisms similar to B36D and S62, which effectively prevent new aggregate formation while breaking down existing fibrils.

### β-syn-36D sequence-based peptide inhibitor restores functional output and normalises ATP amplitude

Mitochondrial function is crucial to the maintenance of cellular homoeostasis. Given that PFF-seeding in primary human fetal neurons resulted in significant downregulation of mitochondrial genes, we used calcium (Ca^2+^) imaging to examine the downstream effects of PFF toxicity and the ability of inhibitor treatment to correct these. We first hypothesised that disaggregating α-syn fibrils could restore the functional outputs caused by PFF accumulation.

**Fig. 5:**
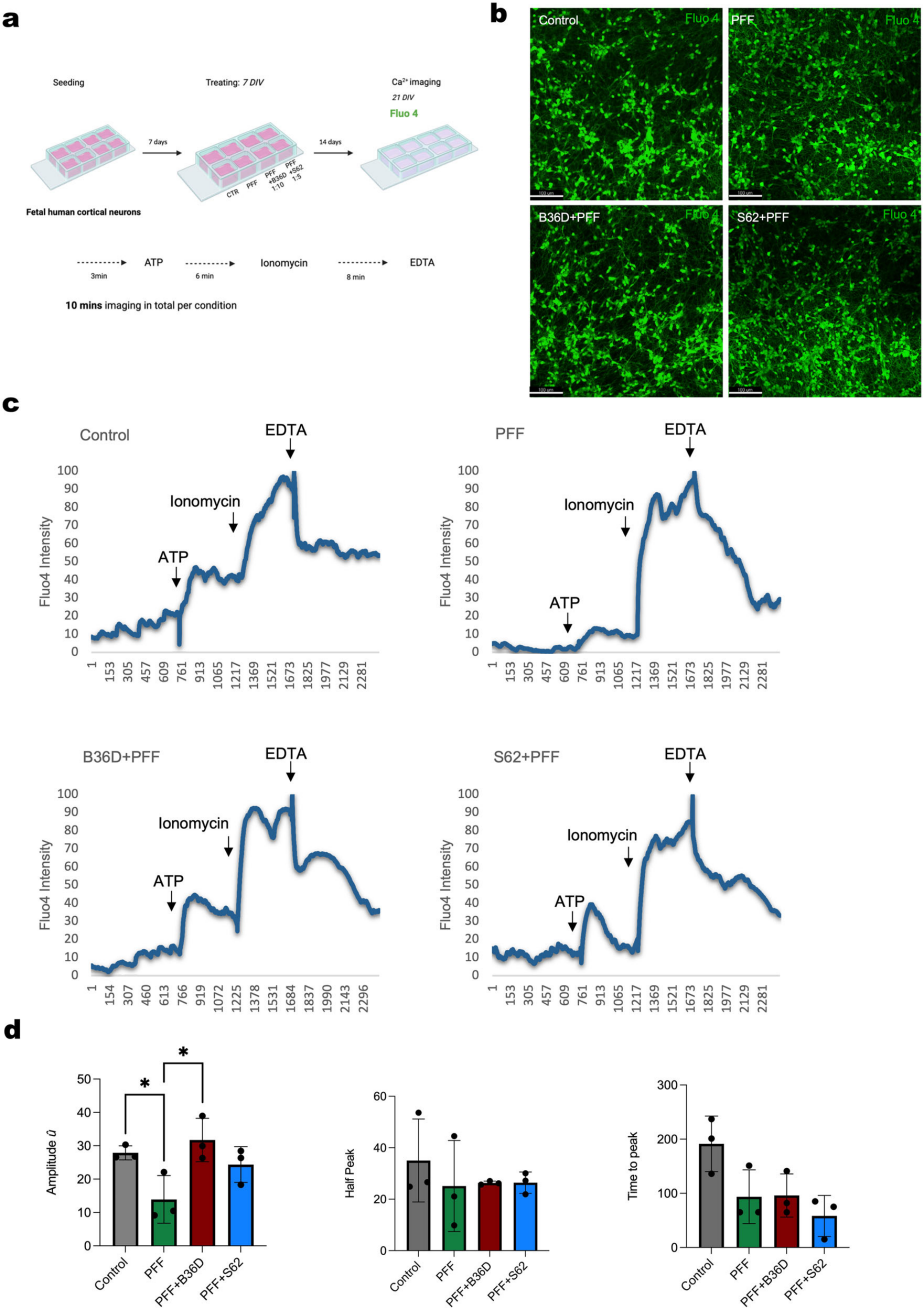
B36D peptide inhibitor restores ATP-induced calcium response in PFF-treated fetal neurons. **a** Schematic of the experimental setup: fetal cortical neurons were seeded with PBS, PFF, PFF: B36D (1:10), or PFF: S62 (1:5) on Day 7. After 14 days of treatment, cells were washed and stained with Fluo-4 for calcium imaging on Day 21. The timeline shows the addition of ATP (100 µM), Ionomycin (10 µM), and EDTA (100 µM) during a 10-min imaging session. **b** Representative Fluo-4 fluorescence images of fetal cortical neurons under each experimental condition. Scale bars, 100 µm. **c** Time course of ATP-induced calcium responses measured by Fluo-4 fluorescence intensity. Values normalised to calibrated fluorescence range (Fmax–Fmin) established by ionomycin and EDTA treatment. The control condition shows technical artefacts at 825 s during ATP addition. Data represent mean ± SD from 40 cells across three independent biological samples. **d** Quantification of ATP-induced calcium response amplitudes, expressed as a percentage change in fluorescence normalised to maximum and minimum signals. PFF treatment significantly reduced response amplitude compared to control, while B36D co-treatment restored responses to control levels. One-way ANOVA with Tukey’s multiple comparisons test revealed significant differences between groups: PFF vs. control (*F*(28.82,14.02) = 4.55, *p* = 0.04), PFF vs. PFF + B36D ((14.02,31.82) = 5.47, *p* = 0.01), but not PFF vs. PFF + S62 ((14.02,24,14) = 3.11, *p* = 0.2). Temporal analysis of Ca^2+^ signalling parameters across experimental conditions. Half-peak duration (measurement of signal width at 50% of ATP amplitude) showed no statistically significant difference between conditions based on paired t-test: PFF vs. Control (*p* = 0.07), PFF vs. PFF + B36D (*p* = 0.92), and PFF vs. PFF + S62 (*p* = 0.903). Similarly, time-to-peak measurements (time required to peak in response to ATP stimulus) showed no statistically significant difference between conditions: PFF vs. Control (*p* = 0.23), PFF vs. PFF + B36D (*p* = 0.79), and PFF vs. PFF + S62 (*p* = 0.34). Data points represent averages from 40 cells per biological replicate (*n* = 3 independent experiments). Each dot represents an independent culture using the same preparation of α-syn PFFs. One batch of PFF is prepared annually, ensuring consistency across experiments, and stored at −80 °C. For each experiment, a new aliquot from storage is utilised, and the PFFs are sonicated immediately before the experiment to maintain their integrity. Each replicate corresponds to a distinct biological human tissue sample, representing independent cultures.

To assess calcium dynamics, we employed the fluorescent calcium indicator Fluo-4 in human fetal cortical neurons. Fluorescence measurements were normalised using a two-point calibration method: maximum fluorescence was established through the application of ionomycin, which facilitates calcium release from intracellular stores and promotes calcium entry at the plasma membrane, while minimum fluorescence was determined following EDTA administration, which chelates extracellular calcium and magnesium (Fig. 5a, b).

Under control conditions, the application of 100 µM ATP to fetal neurons elicited a robust increase in intracellular Ca²⁺ levels, with peaks reaching 60-80% of maximum fluorescence (F/Fmax), demonstrating normal mitochondrial function and calcium handling capabilities. However, neurons treated with PFFs exhibited two distinct abnormalities: (1) reduced spontaneous calcium activity and (2) a significant attenuation of ATP-induced Ca²⁺ influx, with peak responses only reaching 30–40% of maximum fluorescence (Fig. 5c, d). These impairments suggest compromised mitochondrial function and disrupted calcium homoeostasis following PFF treatment. We treated PFF-seeded neurons with the B36D and S62 peptide inhibitors to investigate whether these peptide inhibitors could mitigate this dysfunction. Treatment with B36D produced a striking restoration of calcium signalling, with ATP-induced Ca²⁺ influx amplitudes returning to control levels (60-80% of maximum fluorescence) (Fig. 5c, d). In contrast, S62 treatment led to only a non-significant increase in Ca²⁺ amplitude. Further analysis of Ca²⁺ signalling parameters (half-peak duration and time-to-peak) showed no significant differences across groups (Fig. 5d), suggesting the primary effect of B36D lies in restoring Ca²⁺ signalling strength rather than kinetics. Together, these data propose a mechanistic link between the mitochondrial transcriptomic restoration and functional rescue of calcium homoeostasis, reinforcing the therapeutic potential of B36D in restoring neuronal network function following PFF-induced pathology.

### β-syn-36D normalises gene expression after PFF treatment

**Fig. 6:**
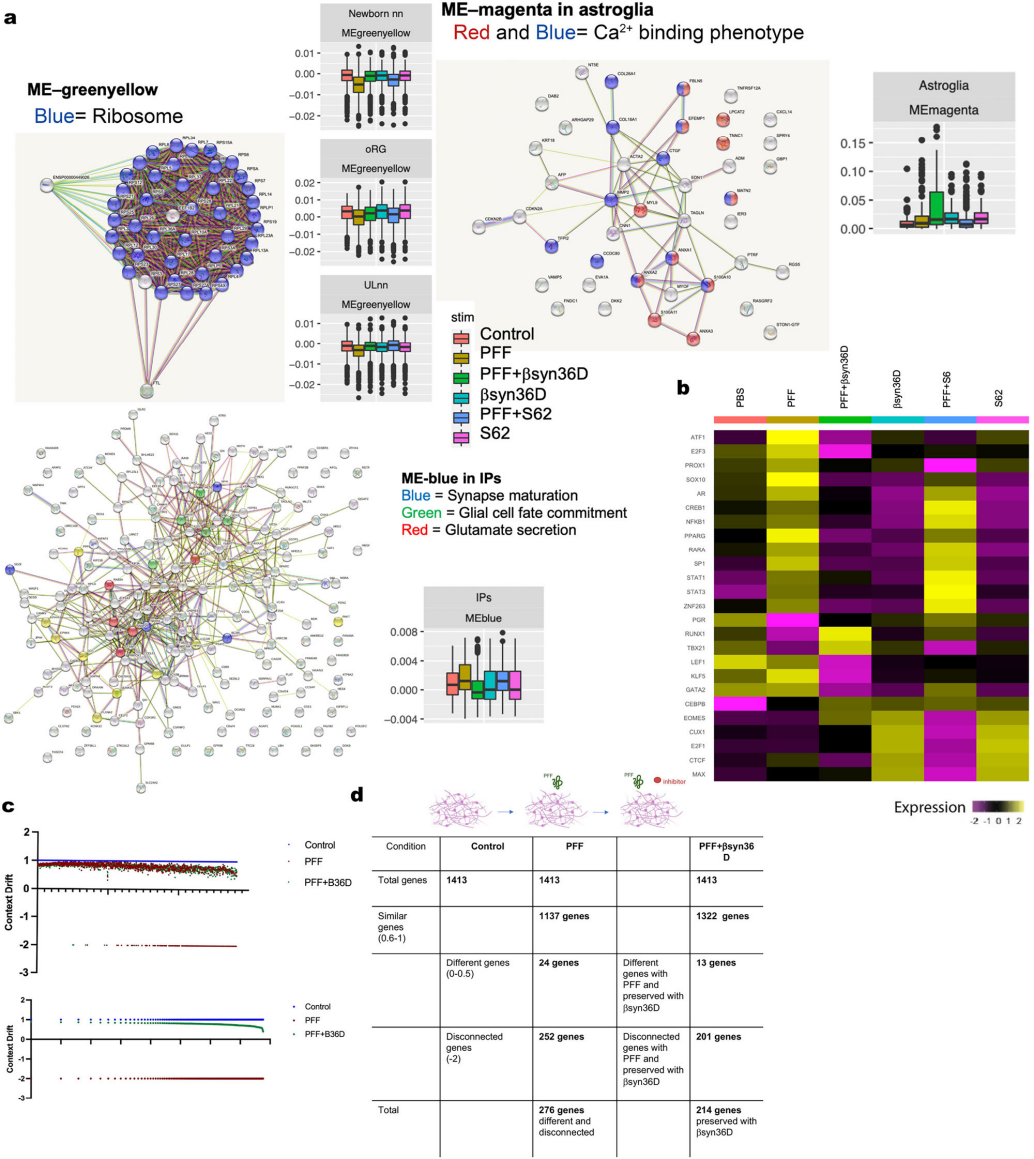
Gene network analysis of transcriptomic differences in the fetal human model seeded with PFF compared to peptide inhibitor treatment groups. **a** Gene ontology analysis based on WGCNA of RNA-seq data from fetal human neurons. Each plot corresponds to a gene module enriched in specific cell types, with dots representing gene expression across six treatment groups: control (orange), PFF (yellow), PFF:B36D (green), B36D (turquoise), PFF:S62 (light blue), and S62 (pink). ME-GreenYellow module: Downregulated in PFF-treated neurons, particularly in newborn neurons, outer radial glia, and upper-layer neurons, with associated ribosomal genes (blue dots). ME-Magenta module: Upregulated in the PFF: B36D group, particularly in astroglia, with genes linked to calcium-binding phenotypes (red and blue dots). ME-Blue module: Upregulated in PFF-treated intermediate progenitors, with genes associated with synapse maturation (blue dots), glial cell fate commitment (green dots), and glutamate secretion (red dots). **b** Heatmap showing transcription factor (TF) analysis of top genes (e.g., ATF1-ZNF263). Yellow represents genes restored to normal expression levels by PFF: B36D treatment. **c** Plot based on gene correlation analysis using a novel approach (see “Methods”). The upper plot represents all the genes: Approximately 252 genes in the PFF group (red dots, at −2) were disconnected and drifted from forming gene communities observed in the control group. Control (blue dots), PFF (red dots), PFF + B36D (green dots). The lower plot focuses specifically on the 252 genes that drifted in the PFF group (red dots, at −2) and illustrates how the B36D treatment (green dots) restored their connectivity, shifting them closer to the control condition (blue dots). **d** Table summarising gene network preservation: In the Control group, 1413 genes were analysed. 1322 genes in the PFF + B36D condition showed a similar correlation to the control (0.6–1), while only 1137 genes in the PFF condition maintained a similar correlation to the control (0.6–1). In the PFF group, 24 genes shifted into different communities compared to the control (0–0.5), while 252 genes completely drifted and disconnected (−2). In the PFF + B36D group, 13 of 24 different genes and 201 of 252 drifted genes were preserved, maintaining closer similarity to the control network.

We then sought to investigate whether these peptide inhibitors counteracted the transcriptomic changes induced by PFF treatment. Single-cell RNA sequencing (scRNA-seq) of human fetal neurons, combined with weighted correlation network analysis (WGCNA), revealed significant insights into the differential gene expression profiles. These findings were consistent with our scRNA-seq analyses, reinforcing the robustness of the results. The ‘greenyellow’ module, predominantly comprising ribosomal genes, exhibited a marked reduction in eigengene expression in outer radial glia (oRG), newborn neurons, and upper layer neurons (ULnn) in PFF-treated samples compared to controls (Fig. 6a). Treatment with B36D effectively restored eigengene expression levels in these cell types, suggesting a reversal of PFF-induced transcriptomic dysregulation. Notably, the ‘magenta’ module, associated with a calcium-binding phenotype, was significantly increased in astroglia in the PFF + B36D condition, highlighting the peptide’s role in modulating calcium-related gene expression. Additionally, the ‘blue’ module, which was elevated in PFF-treated cells and linked to intermediate progenitors, was normalised following B36D treatment. Gene ontology (GO) analysis of this module revealed functional enrichment in pathways related to synapse maturation, glial cell fate commitment, glutamate secretion, and axon guidance (Fig. 6a).

To assess the functional significance of the observed transcriptomic changes, we performed a transcription factor (TF) activity inference analysis. The heatmap displays the average TF activity level for each sample, highlighting the most significantly altered TFs. Notably, among the TFs upregulated following PFF seeding, *ATF1* and *PPARG*—both involved in the heat shock response to protein misfolding—were strongly activated. Interestingly, while these genes were upregulated in the PFF condition, they were normalised in the peptide inhibitor treatment groups PFF + B36D and PFF + S62 (Fig. 6b). Further analysis revealed that six of the top 13 upregulated TFs—*CREB1*, *RARA*, *SP1*, *STAT1*, *STAT3*, and *ZNF263*—were significantly elevated under PFF conditions but were restored to baseline levels with B36D treatment. GO analysis (GO:0032870) confirmed that these genes are primarily associated with the cellular stress response. In contrast, the S62 peptide inhibitor resulted in partial transcriptomic normalisation, with changes observed in transcription factors such as *ATF1*, *SOX10*, and a few other genes, under PFF + S62 treatment conditions (Fig. 6b).

### Gene context and connectome restoration by β-syn-36D in human fetal neurons

To further explore the transcriptional landscape of our scRNA-seq data, we employed REsistance through COntext DRift (RECODR)^22^, a novel method developed to understand phenotypic changes as they relate to gene context between conditions. This method aimed to determine whether PFF treatment significantly changed gene context and whether our peptide inhibitors would cause gene context to resemble normality. The study revealed profound alterations in the gene connectome induced by PFF treatment and highlighted the efficacy of the B36D peptide inhibitor in reversing these changes.

We asked if control fetal neurons maintained the same surrounding gene context patterns after PFF seeding or whether PFF treatment disrupted these networks. The 10x scRNA-seq dataset revealed that, under control conditions, 1413 genes formed a well-defined gene community with established connectivity patterns. However, following the PFF treatment, 252 of these genes exhibited significant alterations in gene context, indicating a disruption in their connectivity within the original network, as shown in the plot by red dots at (−2) in the PFF condition (Fig. 6c). The bottom plot provides a zoomed-in view of these 252 genes (red dots, −2), clearly illustrating how B36D treatment (green dots) prevented their displacement, although there were still a subset of genes whose cosine similarities were lower (<0.6) suggesting that their surrounding environment had not completely returned to a normal state. Nevertheless, this observation suggests a shift towards a more control-like condition with the prevention of a full-blown PFF-induced molecular signature. Additionally, 24 genes in the PFF condition exhibited lower cosine similarity, suggesting their integration into a network community distinct from the control (Fig. 6d).

The 252 genes that underwent connectivity changes in the PFF condition have been insufficiently characterised in the literature, necessitating further investigation to understand their roles, as these may unmask novel genes involved in the pathogenesis of PD. As illustrated in the supplemental data (Supplementary Fig. 2c), these genes are involved in cell cycle regulation and various other regulatory pathways. Their selective drift under PFF conditions may reveal important mechanistic insights into the cellular response to PFF treatment.

Following treatment with B36D, 201 out of 252 genes (79%) that were disconnected in the PFF condition (indicated by a score of –2) were restored on the graph, exhibiting cosine similarity values greater than 0.7. This suggests that B36D treatment was able to shift these genes toward a more control-like transcriptional state, indicating that the peptide inhibitor can largely prevent the molecular disruptions associated with PFF exposure (Fig. 6c, d). Additionally, 13 out of 24 genes (54%) that had shown low similarity to the control (cosine similarity <0.5) under the PFF condition regained strong connectivity with their surrounding genes following B36D treatment, reaching cosine similarity values above 0.7 (Fig. 6c, d). This restoration of network topology strongly suggests that B36D drives a significant reversion toward a normal cellular phenotype by re-establishing critical gene-gene interactions that are disrupted by pathological protein aggregation. The high percentage of reconnected genes implies that B36D targets the fundamental pathways necessary for maintaining cellular homoeostasis, effectively counteracting the pathological cascade initiated by PFF exposure. These findings present compelling evidence that therapeutic interventions targeting protein-protein interactions can not only halt disease progression but potentially reverse established molecular pathologies by restoring normal cellular architecture and signalling networks.

While Differential Gene Expression (DGE) in Fig. 2 identified the most severely impacted individual genes (Mitochondrial and Ribosomal pathways), we utilised REsistance through Context Drift (RECODR) analysis in Fig. 6 to determine if B36D treatment restored the global network architecture. RECODR is an agnostic, network-based approach that maps transcriptional remodelling by assessing changes in gene context. This unbiased analysis demonstrated that B36D successfully reversed the pathology in the network context, with approximately 79% (201 out of 252) of dysregulated genes shifting toward the normal healthy state.

## Discussion

The pathophysiological understanding of PD has been significantly limited by the absence of models that faithfully recapitulate the disease. While α-syn aggregation and LB formation are hallmarks of PD, a comprehensive model system that accurately replicates human PD pathology and provides reliable therapeutic testing platforms is still lacking.

Early cellular models, such as human embryonic kidney (HEK) cells, provided initial insights into α-syn aggregation and toxicity^23^. Primary rodent neurons offered improved physiological relevance and have enabled the study of α-syn pathology within neuronal networks^15,24,25^, Yet, these models fail to accurately represent human pathology, leading to numerous failed clinical trials despite promising preclinical results. Animal models have presented their own significant limitations. Notable among these is the observation that α-syn overexpressing animals often fail to develop authentic LB-like aggregates.

The advent of human pluripotent stem cell (hPSC) technology appeared to bridge some of these gaps by enabling the generation of patient-specific neurons^26^. The successful application of CRISPR/Cas9n technology to generate SNCA+/− and SNCA−/− cell lines demonstrated the potential for creating disease-resistant midbrain dopaminergic neurons^27^. However, hPSC-derived neurons present their own challenges, including potential genetic alterations from clonal selection and culture conditions, limiting their utility for therapeutic development.

In addressing these limitations, our study employed primary human fetal neurons as an in vitro model, offering several distinct advantages. These neurons provide a more physiologically relevant platform compared to hPSC-derived neurons, as they better represent the cellular environment of the normal human brain. Their stable genetic background, free from the variability inherent in hPSC-derived neurons, makes them particularly valuable for studying α-syn pathology. This stability is especially crucial for scRNA-seq analysis following PFF treatment, enhancing the reliability of gene expression data and allowing for more confident interpretation of any changes.

B36D is a sequence-based peptide derived from β-synuclein, previously reported to inhibit α-syn aggregation^19^. This peptide, designed to target the N-terminal region (amino acids 36–45) of α-syn, obstructs misfolding by disrupting aggregation-prone interactions^19^. In this study, we evaluated the ability of B36D to inhibit α-syn PFF aggregation in a human neuronal model. Numerous studies have highlighted the critical role of the NAC region of α-syn in fibril formation and pathology^28–31^, The NAC region promotes endogenous α-syn to adopt a zipper-like configuration, driving fibril propagation. S62, a structure-based peptide inhibitor, was designed to target the tip of the steric zipper protofilament, effectively capping fibril elongation^20^.

In this study, we have described a novel model in which relevant intracellular PD pathology in the form of α-syn aggregation is recapitulated in human cortical neurons. Furthermore, we observed downstream pathology, with mitochondrial gene dysregulation and dysfunctional oxidative phosphorylation following PFF treatment, all of which could be prevented by disruption of α-syn aggregation, using peptide inhibitors. Both our sequence-based (B36D) and structure-based (S62) peptide inhibitors were highly effective in disaggregating α-syn fibrils, both in vitro and in cell-free conditions, and prevented further aggregation. Importantly, prevention of α-syn aggregation abrogated the downstream toxic effects, indicating a potential therapeutic approach that warrants further exploration. Although the potential toxicity of intermediate α-syn species is a concern in any α-syn targeting strategies, we did not see any toxicity related to intermediate species generated during fibril breakdown.

B36D and S62 were found to disrupt mature α-syn aggregates, as evidenced by reduced pFTAA signal and altered fibril morphology under TEM. However, it is possible that B36D more effectively promotes the formation of non-toxic α-syn conformers compared to S62, a hypothesis warranting further investigation using cryo-EM or other high-resolution structural approaches. Alternatively, the phenotypic improvements observed with B36D may arise from mechanisms independent of aggregate disassembly.

While previous studies^19,20^ suggested that modified versions of these peptides may exhibit resistance to proteolysis, the specific stability and half-life of the native peptides used here were not directly measured in our system. Consequently, we cannot exclude the possibility of peptide degradation over the 10-day treatment period. Nevertheless, the robust functional and transcriptomic rescue observed suggests that the peptides remain sufficiently stable to exert a protective effect during the critical early stages of PFF-induced seeding. Whether this protection stems from sustained intracellular presence or a transient ‘hit-and-run’ extracellular effect remains to be determined. Indeed, since both B36D and S62 have been shown to disaggregate α-syn fibrils in vitro, co-incubation may have reduced PFF uptake by breaking down fibrils extracellularly, thereby lowering intracellular burden. Thus, while our findings confirm that the net reduction of toxic aggregates effectively preserves neuronal health, the current simultaneous-treatment design does not allow us to definitively distinguish between extracellular and intracellular actions. Therefore, the functional recovery may involve potential intracellular modulation in addition to the prevention of uptake, and future experiments involving sequential rather than simultaneous application of PFFs and peptides will be needed to disentangle whether inhibitory activity occurs primarily within neurons or in the extracellular milieu.

A major strength of our study lies in the comparison between our fetal neuron model and postmortem PD datasets, highlighting the physiological relevance of our findings. The dysregulation of mitochondrial pathways observed in our model aligns with mitochondrial dysfunction reported in postmortem PD samples, including reduced complex I activity and oxidative stress^32,33^, The ability of our model to replicate these patterns validates its utility as a preclinical platform.

The relationship between α-syn aggregation and mitochondrial dysfunction remains a cornerstone of PD research, including genetic studies. Consistent with prior studies^34,35^, our findings suggest that α-syn aggregation precedes and contributes to mitochondrial dysfunction. This is evidenced by the dysregulation of mitochondrial gene expression and oxidative phosphorylation in our model, a hallmark also observed in PD postmortem tissue.

The N-terminal mitochondrial targeting sequence of α-syn is hypothesised to mediate its interaction with mitochondrial membranes, promoting aggregation at these sites^34^. Additionally, transgenic mouse models expressing aggregation-prone α-syn variants (A53T and A30P) demonstrate accumulation of mitochondrial DNA damage, compared to wild-type controls^32^, indicating that mitochondrial dysfunction in PD, occurs downstream of α-syn aggregation in a pathogenic pathway. In neuronal cell models, α-syn overexpression has also been shown to result in pathological mitochondrial alterations, including morphological changes, reduced activity, and elevated free radical production^35–37^,

While we are highly constrained by the scarcity of primary human fetal cortical tissue, we were able to perform an independent bulk RNA-sequencing experiment on a separate batch of fetal tissue to specifically test the effect of monomeric α-syn. This *n* = 1 bulk RNA-seq study compared the transcriptional profile of neurons treated with α-syn PFFs vs. those treated with monomeric α-syn, and we observed the clear and significant down-regulation of the mitochondria genes in the PFF-treated condition (Fig. 2d) (mirroring the pathology seen in our scRNA-seq data), but crucially the monomeric α-syn condition showed no comparable pattern of transcriptional dysregulation.

Of further interest with our model is the use of new computational approaches. In particular, RECODR revealed a fascinating phenomenon where 252 genes completely drifted in the PFF-treated condition, forming new connections with surrounding genes (Fig. 6c). This dramatic rewiring of the gene interaction network suggests a substantial cellular response to PFF treatment. Notably, treatment with the B36D inhibitor (PFF + B36D condition) prevented this network reorganisation, maintaining these genes in positions similar to those found in the control condition. These 252 critical genes, primarily associated with adherens junction formation and cell cycle regulation pathways (Supplementary Fig. 2c), represent an understudied aspect of PD pathophysiology.

Our model, while providing valuable insights, has a number of limitations. First, we saw no cell death or synapse loss following α-syn PFF seeding. This might reflect insufficient incubation time with PFFs, as our observations were limited to 14 days due to technical constraints. Extending the observation period presents significant challenges, as prolonged culture conditions can lead to cell detachment and compromised viability, making it difficult to differentiate between PFF-induced effects and culture-related artefacts. In relation to this, the discrepancy between the stable metabolic activity (PrestoBlue) and the significant reduction in cell number (DAPI) at Day 14 likely reflects the different sensitivities of these assays in a heterogeneous primary culture (Supplementary Fig. 1h, i). While DAPI provides a definitive readout of irreversible cell loss, bulk metabolic assays like PrestoBlue can be confounded by compensatory metabolic activity from surviving, resilient populations such as astroglia. Our high-resolution transcriptomic and functional data, showing widespread mitochondrial gene downregulation and impaired Ca^2+^ dynamics, confirm that the model indeed exhibits progressive neurodegenerative stress prior to the point of structural cell loss. Secondly, the cells used are fetal in origin and not aged. The culture lacks microglia and oligodendrocytes, which may be integral to PD pathogenesis in adult brains. Interestingly, we did find an increase in astrocyte numbers after PFF treatment and this may represent an intrinsic protective response against α-syn related pathology. Finally, we studied only one CNS region- cortex- and the disease process may be different at other sites such as the nigra.

In conclusion, our study establishes a physiologically relevant human fetal neuron model that successfully recapitulates key aspects of PD pathology, particularly α-syn aggregation and subsequent mitochondrial dysfunction. The model’s validity is strengthened by its alignment with post-mortem PD tissue findings, especially regarding mitochondrial pathway dysregulation and our demonstration that peptide inhibitors can effectively prevent α-syn aggregation and its downstream effects, showing causality. While the model has certain limitations, including the age of cells, the absence of cell death and limited cellular diversity, it offers significant advantages over existing platforms through its stable genetic background and physiological relevance. The successful replication of disease-relevant phenotypes, combined with the model’s utility in therapeutic testing, positions this system as a valuable tool for both mechanistic studies and drug development in PD research. Future refinements incorporating extended observation periods and multiple cell types will further enhance its applications in understanding PD pathophysiology and developing effective treatments.

## Methods

### Primary human cells

Human fetal tissue was collected from routine termination of pregnancies at Addenbrookes Hospital, and experiments were performed at the John van Geest Centre for Brain Repair, University of Cambridge. All human tissue was collected under full ethical approval in accordance with the United Kingdom’s Department of Health guidelines and local ethical approval (NRES Committee East of England- Cambridge Central, reference no. 96/085). Primary human brain tissues were dissected by a senior technician within the group and placed into a Hibernate-E medium (Thermo Fisher Scientific, A1247601). Fetal human cortical was dissociated with Accutase (Sigma-Aldrich, A11105-01) for 30 min at 37 °C, centrifuged for 5 min at 1000 rpm, washed in DMEM (Thermo Fisher Scientific), and resuspended in DMEM media using a P100 pipette. Cells were plated on glass coverslips coated with poly-D-lysine and 1 µg/mL laminin; plated cells were cultured in DMEM media supplemented with 2% B-27^TM^ (Thermo Fisher Scientific, 17504044), 1% bovine serum albumin (BSA) (Thermo Fisher Scientific, 10500064), 1% antibiotic-antimycotic (PSF). Cells were then plated onto the coated glass coverslips in a 24-well plate at a density of 40,000 cells/cm^2^ or in a 6-well plate at a density of 2,000,000 cells/cm^2^ and incubated in a 5% CO_2_ incubator in the containment level two (CL2) laboratory.

### Preparation of recombinant α-synuclein pre-formed fibrils

α-Syn preformed fibrils (PFFs) were generated by dissolving wild-type human recombinant α-syn monomers (rPeptide, S-1001-2) and then resuspending them in MiliQ H2O. An Amicon® Ultra 3 K device (Merck, UFC200324) was pre-rinsed with MiliQ H2O, which was then placed on a rotor and centrifuged at 3600 × *g* for 60 min. After centrifugation, the Amicon® Ultra filter device was separated from the filtrate collection tube. To recover the concentrated solute, the Amicon® Ultra filter device and the concentrate collection tube were inverted and centrifuged for 2 min at 1000 × *g* to transfer the solute from the device to the collection tube. The concentrated α-syn was resuspended in 10 mM Tris-HCl, pH = 7.6, 50 mM NaCl, to a 5 mg/mL concentration. The α-syn-containing tube was then placed in a 37 °C thermomixer and shaken for 7 days at 1000 rpm. After 7 days, PFFs were aliquoted into 20 μL lots containing 5 μg/μL and immediately stored at −80 °C. Prior to use, stock PFF was diluted 1:50 in DMEM medium. This working solution of PFFs was then sonicated for 60 min in a water bath sonicator (Decon, FS100b) before the desired volume was added to the wells. On day 7 in vitro (DIV), a half-medium change was performed, and then, following the half-medium change, PFFs were added at a final concentration of 70 nM. For PFF seeding, fetal human cortical neurons were cultured either in microfluidic chambers (used only for the compartmentalised uptake assay) or in standard culture plates (used for all biochemical, imaging, and transcriptomic experiments). On DIV7, α-syn PFFs were added directly to the culture medium without transfection reagents, allowing uptake to occur through endogenous cellular pathways and more closely mimic physiological mechanisms of aggregate internalisation observed in vivo. Uptake was confirmed by confocal microscopy, which revealed discrete α-syn puncta localised within neuronal somata and processes, consistent with intracellular internalisation. These methodological precautions ensured that all downstream imaging, biochemical, and transcriptomic analyses reflected genuine intracellular PFF-induced pathology rather than residual surface-bound fibrils.

### Preparation of fluorescent-tagged α-synuclein PFF with Atto 594

Conjugating maleimide Atto-594 to α-syn typically involves a thiol-maleimide reaction, forming a stable thioether bond. Notably, at pH = 8.5, maleimide preferentially reacts with primary amines, allowing for an alternative method to label PFF. This protocol does not alter the seeding properties of PFF. Briefly, 1 mg of 594 maleimide dye (MW 1358 g/mol) (Merck, 08717) was dissolved in 1 mL of PBS. The pH was adjusted to 8.5, and then 9.43 μL of the dissolved dye in PBS was added to 20 μL of α-syn PFF to have a final concentration of 250 μM. The tagged PFF was incubated for 2 h at room temperature (RT) in the dark. The tagged PFFs were ultra-centrifuged at 100,000 × *g* for 1 h at 4 °C. The supernatant was collected, and the pellet was re-suspended in 20 μL of PBS. The washing steps were repeated until the excess was removed. The tagged PFFs were stored at −80 °C.

### Sequence-based peptide inhibitors β-synuclein 36D inhibitor

A published protocol was available for the preparation of the peptide inhibitor retro-inverso βsyn 36D^19^. However, the procedure was optimised to make it more suitable for in vitro work. The amino acids, which retro-inverso βsyn36D consisted of: (dR)(dT)(dK)(dS)G(dV)(dY)(dL)(dV)G, were purchased (ProteoGenix, GMPT59-98). These amino acids were mixed with 12 mL of solvent solution (Sodium Sulfate 142.04 g/mol, 1.42 g and Sodium Phosphate 142.99 g/mol, 357 mg) and then filtered with a 0.2 µm filter to give a final concentration of 70 μM. It was then aliquoted and stored at −80 °C.

### Structure-based peptide inhibitors S62

S62 has been used according to a designed protocol. The amino acids, which S62 consisted of: GAVVWGVTAVKKGRKKRRQRRRPQ, were purchased (ProteoGenix, GMPT59-98) and mixed with 47.5 mL of the solvent solution (Sodium Sulfate 142.04 g/mol, 1.42 g and Sodium Phosphate 142.99 g/mol, 357 mg). The solution was then filtered with a 0.2 µm filter to give a final concentration of 70 μM and aliquoted and stored in a −80 °C freezer.

For inhibitor experiments, the peptide inhibitors (B36D and S62) were added immediately after PFF application, and the cultures were then incubated for a further 14 days. To minimise extracellular artefacts and prevent non-specific membrane binding of PFFs, the culture medium DMEM media supplemented with 2% B-27^TM^ (Thermo Fisher Scientific, 17504044), 1% bovine serum albumin (BSA) (Thermo Fisher Scientific, 10500064), 1% antibiotic-antimycotic (PSF) was refreshed every 7 days during the 14-day incubation period to ensure removal of any residual extracellular material. Both PFFs and inhibitors were applied once at DIV7, with inhibitors added directly to the culture medium immediately after PFF addition, without pre-mixing.

### Seeding human fetal cortical neural cells in microfluidic chambers

The imaging dishes (MATTEK, P50G-0-30-F) were coated with 1 mL of 2 mg/mL PDL overnight at 4 °C and labelled with an arrow to indicate the top of the dish. Dishes were washed twice with sterile water on the day of culture and left in the hood to dry before attaching the microfluidic chamber (Xona microfluidics, SND 150). Matrigel (Thermo Fisher Scientific, 11553620) was diluted 1:200 in cold DMEM high glucose. For both top and bottom compartments, 150 μL was added into all wells, making sure there was flow through the chamber into the other well and then left for 1 h at 37 °C in the incubator. Matrigel was removed from the chambers and 10 μL of cells loaded into the microgroove only on one side of the top well. Two to four hours was allowed for the cells to attach. No cells were seeded in the bottom wells. Finally, chambers were filled up with DMEM medium supplemented with 2% B-27™ (Thermo Fisher Scientific, 17504044), 1% BSA (Thermo Fisher Scientific, 10500064), and 1% PSF, 400 μL in the top compartment and 300 μL in the bottom compartment (see illustration in Fig. 1a). Microfluidic chamber experiments (*n* = 3 biological replicates) were performed to assess uptake of fluorescently tagged α-syn PFF by human fetal cortical neurons. Experimental groups included control, 1-day PFF treatment, and 7-day PFF treatment. Cells were treated at DIV14.

### Live imaging

Were performed on human fetal cortical neurons (*n* = 5 biological replicates). Cells were treated at DIV7 with control (PBS), α-syn PFF (70 nM), or PFF plus peptide inhibitor, and stained with pFTAA (4 μM) 1:1000 (Merck, SCT066) to visualise filamentous α-syn. pFTAA stains filamentous protein species, including α-syn^38^. The PFF-treated cells were incubated at 37 °C for 30 min. The Essen Incucyte Zoom (objective 20x) was used for long-term imaging. Cells were maintained in a 5% CO_2_ incubator, and imaging was captured every 8 h for 14 days.

### Immunocytochemistry: sample preparation and staining

Cells were fixed in 4% paraformaldehyde (PFA) for 15 min at RT. All samples were then washed three times in PBS. A blocking solution was then applied for an hour at RT and consisted of 3% donkey serum and 0.3% Triton X-100 in PBS. Primary incubations were performed overnight at 4 °C, followed by three PBS washes, and secondary incubation was performed as 2 h incubations at RT. Finally, the plated cells were washed three times with PBS. Antibody solutions contained 1% donkey serum and 0.3% Triton X-100 in PBS.

### Image acquisition and processing

Images were acquired using a Leica Stellaris 8 confocal microscope (*z*-stack step size: 0.1–1 μm, 20x–63x objective, 1024 × 1024-pixel images, 10–20 frame averaging). Laser power, camera exposure and gain were kept the same whilst acquiring images for each experiment. Confocal image quantification was performed using the CellProfiler software (v.3.1.9; http://cellprofiler.org). Non-confocal images were acquired using a fluorescence microscope Cellomics™ Array Scan XTIn (Thermo Fisher Scientific) and ×20 or ×40 magnification. Laser power, camera exposure and gain were kept the same whilst acquiring images for each experiment. For any replicate staining, these settings were also kept the same. For Cellomics™ image quantification, all analysis was performed using the Cellomics™ software Neuroprofiler programme.

#### Automated analysis of pFTAA internalisation and neuronal morphology

Cellular uptake of pFTAA and morphological analysis was performed using Cellomics™ Neuroprofiler and Spot Count automated software. Colocalisation of pFTAA-positive signals with neuronal markers (MAP2 and TUJ1) was quantified using the Spot Count analysis module. Both spot count and signal intensity were measured. Morphological analysis was conducted using the Neuroprofiler software’s automated masking system. Nuclear regions were identified using DAPI staining, while neuronal somata and processes were detected using neural-specific antibodies. Mean cell body area was calculated from DAPI-positive regions associated with a neuronal marker mask. Neurite length was measured using the neural marker-positive processes mask extending from identified cell bodies, and mean neurite length was calculated for each condition. Colocalisation analysis of pFTAA-positive PFF with neuronal markers MAP2 and TUJ1 was performed on human fetal cortical neurons (*n* = 5 biological replicates, with duplicates per condition). Experimental groups included control (PBS), PFF treatment on day 1, day 7, and day 14 post-treatment. Mean intracellular spot counts of pFTAA+ aggregates co-localised with neuronal markers were normalised to DAPI to account for cell number.

#### Synapses count and intensity analysis

Synaptic quantification was performed using the Cellomics™ Spot Count analysis software. The system measured both the number and intensity of synaptic markers, synaptophysin-1 and vGLUT1 were used. Experiments were conducted with *n* = 3 biological replicates for each marker, with experimental groups including control (PBS) and PFF treatment on day 1, day 7, and day 14 post-treatment. Spot detection parameters were optimised to identify discrete synaptic puncta, enabling quantification of synaptic density and relative protein expression levels.

### Protein extraction and immunoprecipitation of α-synuclein

Cell preparation and lysis: human fetal cortical neural cells (2,000,000 cells/well) were cultured in 6-well plates. After PBS washing, cells were lysed in cold Triton X-100 buffer (150 mM NaCl, 50 mM Tris HCl pH 8.0, 1% Triton X-100) containing protease (Roche, 3115887001) and phosphatase (Roche, 4906845001) inhibitor cocktails. Cells were scraped (Thermo Fisher Scientific), rotated for 15 min at 4 °C, and homogenised through a 21 G needle (20 passes). Following another 15-min rotation at 4 °C, lysates were centrifuged (10,000 × *g*, 15 min), and supernatants were collected. Protein concentration was determined using a BCA assay (Thermo Fisher Scientific, 23227).

Immunoprecipitation: samples were diluted to 0.5–2 mg total protein in 500–800 μl lysis buffer with inhibitors. Input samples (5–10%) were reserved. α-Syn antibody (MJFR1, Abcam 138501; 1:600) was added for overnight incubation at 4 °C. Dynabeads™ (50 μL/reaction; Thermo Fisher Scientific, 10007D) were washed with Ab Binding and Washing Buffer and resuspended in supplemented lysis buffer (50 μL beads/105 μL buffer). Beads (100 μL) were added to each sample and incubated (30 min at 4 °C, 10 min at room temperature). After washing, beads were transferred to new tubes and protein was eluted using 30 μL lysis buffer containing NuPAGE LDS Sample Buffer and Reducing Agent (1X).

Western blot analysis: samples were heated (70 °C, 10 min) and loaded onto 4–12% Bis-Tris gels (Thermo Fisher Scientific, NP0335BOX) alongside Kaleidoscope standards (Bio-Rad, 161-0377). Proteins were separated (80–120 V, 2 h) in MOPS-SDS buffer (Thermo Fisher Scientific, NP0001) and transferred to PVDF membranes (GE Healthcare, 10600023) overnight at 15 V, 4 °C in transfer buffer (425 mL H_2_O, 25 mL NuPAGE Transfer Buffer 20x, 50 mL methanol, 250 μL SDS 20%).

Immunodetection: membranes were fixed (4% PFA, 30 min), washed (TBS-T, 6x), and blocked (5% milk, 1 h). Primary antibody (α-syn syn42, 1:1000–2000, BD Biosciences, 610787) was applied overnight at 4 °C, followed by HRP-conjugated secondary antibody (1:1000, Abcam 131368, 1–2 h). Detection used SuperSignal West Pico substrate (Thermo Fisher Scientific, 34080) on an UVITEC system (Cleaver Scientific). Input membranes were stripped (15 g glycine, 1 g SDS, 10 mL Tween 20, pH 2.2), washed, and probed for β-Actin before ECL™ detection (Thermo Fisher Scientific, 34080). Western blot analysis was performed on human fetal cortical neurons (*n* = 5 biological replicates). Experimental groups included control (untreated) cells, PFF-treated cells as pathological control, and cells treated with PFF in combination with peptide inhibitors at different ratios.

### Fluorescence microplate reader assay

The Fluostar Omega (BMG LABTECH) was used to read all fibrils in a 96-well black plate (Thermo Fisher Scientific, 165305). The number of cycles was set to 1000 cycles at 37 °C and a reading was taken every 15 min for 2 days, and no shaking was required. Samples were taken from these fibrils at different time points and stored at −80 °C for imaging. Fluorescence microplate reader assays were performed on fibrils (*n* = 3 biological replicates). Experimental groups included control, PFF-treated, and PFF combined with peptide inhibitors at different ratios.

### Transmission electron microscopy (TEM)

Following the fluorescence microplate reader assay (2.9) the fibril samples were prepared for TEM imaging (Tecnai G2 80–200 keV). Sucrose fractions were added to glow-discharged, holey carbon grids (EM Resolutions, 400 mesh Copper) for 1 min. Afterwards, they were washed with distilled water twice for 30 s and stained with uranyl acetate (1%, w/v) for 1 min. Images were acquired under minimal dose conditions with a Tecnai G2 transmission electron microscope at 200 kV. Images were taken at ×14,500–×19,000 magnification. Length measurement and quantifications were performed using ImageJ analysis software.

### Calcium imaging

Cell preparation: human fetal cortical neural cells were seeded in ibidi chamber μ-Slides (ibidi, 80826; 25,000 cells/well). Calcium imaging experiments were performed on human fetal cortical neurons (*n* = 3 biological replicates). Experimental groups included control (PBS), PFF treatment (70 nM), and PFF combined with peptide inhibitors β-syn36D (1:10) or S62 (1:5); treatment was added at 7 DIV.

Calcium indicator loading: after 14 days of PFF and inhibitor treatment, cells were stained with Fluo4 (Thermo Fisher Scientific, F14201). The indicator was prepared as 1 mM stock in DMSO (Sigma-Aldrich, D2650) and diluted 1:100 in calcium imaging media (HBSS without phenol [Gibco, 14065056] supplemented with B-27™ [1:50, Thermo Fisher Scientific, 17504044]). Cells were incubated for 1 h at 37 °C, washed three times, and maintained in imaging media for an additional hour before imaging.

Image acquisition: live imaging was performed using an Andor Dragonfly spinning disk confocal microscope (20x objective) with images captured every 300 ms. ATP (100 mM; Thermo Fisher Scientific, R0441), ionomycin (5 mM; Thermo Fisher Scientific, 124222), and EDTA (10 mM) solutions were added at 3.5-min intervals during imaging. Ionomycin and EDTA were used to establish maximum and minimum fluorescence signals, respectively.

Data analysis: Fluo4 fluorescence intensity was calculated as a percentage using the formula: ((F − Fmin) × 100)/(F − (Fmax − Fmin)) where Fmax represents maximal fluorescence post-ionomycin and Fmin represents minimal fluorescence post-EDTA. ATP response parameters (amplitude, time to peak, and half peak) were analysed. Statistical analyses were performed using GraphPad Prism (Version 9.4.1, macOS), with *p* < 0.05 considered statistically significant.

### Human fetal cortical cells dissociation and preparation for single cell RNA-sequencing (scRNA-seq)

Plated cells were washed with 1x dPBS (Sigma-Aldrich, D8537). The dissociation solution used was 200 μL/well in a 6-well plate of TrypLE (Thermo Fisher Scientific, 12605010) and incubated with the cells at 37 °C for 3 min. Hibernate-E medium (Thermo Fisher Scientific, A1247601) supplemented with 10% FBS (Thermo Fisher Scientific, 10500064) and 2% B-27^TM^ (Thermo Fisher Scientific, 17504044) was added in a ratio (10:1) (Hibernate-E:TrypLE) to inactivate TrypLE. The cells were dissociated by gentle pipetting and filtered using a 50-μm strainer (CellTrics®, 04-004-2327) into a 15-mL Falcon tube. The cells were centrifuged at 500 × *g* for 5 min at room temperature. For scRNA-seq samples (*n* = 1), the cell pellet (20,000 cells per sample except for the negative control conditions B36D and S62 was less) was resuspended in (45 μl per sample) Hibernate-E medium supplemented with 1% FBS, and 2% B-27^TM^ and the suspension was transferred to processing on ice. The single-cell solution was run through the Sequencer NovaSeq 6000 unit, which is available at the sequencing core in Cancer Research UK (CRUK). Each sample was run on one separate lane of an S1 flow-cell type and using the 10X Genomics platform. This platform consists of a droplet-based sequencing method.

For FACS samples, the cell pellet was resuspended in DPBS without calcium and magnesium (Thermo Fisher Scientific, 14190136) containing 0.05% BSA (Sigma, A9418) and directly incubated for an hour with pFTAA and Zombie NIR™ (Biolegend, 423105) at RT. The samples were washed in 0.05% BSA dPBS and centrifuged for 5 min at 500 G. After a final wash, the cells were resuspended at a concentration of approximately 3 million cells per 500 μL. The samples were transferred immediately on ice to the Flow Cytometer unit at the Jeffrey Cheah Biomedical Centre (JCBC). Flow cytometry was acquired using a BD FACSAria^TM^ Fusion Flow Cytometer. Automatic compensation was applied using individually stained markers (pFTAA marker: green laser/filter 525/50-404 nm, Zombie: far red laser/filter 780/60-640 nm), and negative controls and gates were determined using an isotype control. Cells were filtered for doublets and debris.

### scRNA-seq pipeline and analysis

The initial scRNA-seq data-analysis pipeline was generated using the CellRanger 3.1 software package, with reads aligned according to published protocols^39^. CellRanger detected 85% fraction reads on average per cell and 1600–2100 median genes per cell. Bias from false cell discovery due to potential ambient RNA contamination or low UMI counts was eliminated in two samples using the “force cell” function in CellRanger. Data were processed in Seurat 3.1/4.0.1. Counts were loaded into R (version 4.0.3) and filtered based on a minimum of three cells expressing each gene. As a standard method, only those cells were included in the analysis that expressed 200–5000 genes and in which the proportion of mitochondrial genes were below 25% out. Cells displaying a greater fraction of mitochondrial transcripts are conventionally regarded as nonviable and were discarded to avoid bias in the transcriptomic analysis. Clustering was performed in Seurat over 14 dimensions with a resolution of 0.4, and cell type or state identities were determined by the expression of previously defined markers.

For trajectory reconstruction, Monocle 3 (v.0.2.3.0) was used on the entire merged dataset. Cells with a transcriptomic signature of cell stress were removed, resulting in 75,497 processed cells. The top 3000 variable features calculated by Seurat over 100 dimensions were used for the ‘preprocesscds()’ function. DEGs were obtained for each sample (fetal control vs. fetal PFF) and were only retained if significantly different (adjusted *p* value < 0.05) for both individual and combined comparisons. Overlap was then identified between the sample-specific list of DEGs.

WGCNA was used to reveal potentially affected pathways in each cell type in all samples using the top 3000 variable features (WGCNA v.1.70-3). A minimum module size of 15 and a deep split of 4 were retained. The resulting modules were then projected onto the merged dataset using ‘moduleEigengenes()’. Significant differences between module eigengene values for each sample are presented in box plots. Overlap between genes from each module and DEGs calculated for the cell type was then plotted using Python.

Additionally, STRING (https://string-db.org) was utilised for network interaction analysis (‘medium confidence’), and the disconnected nodes were eliminated. Finally, enrichment analysis was performed for gene sets included in the networks of highly correlated genes by GO analysis via the EnrichR platform. The –log() of adjusted *p* values or the false discovery rate was taken to generate clustermap plots.

To infer potential functional relevance for differentially affected genes in the PFF sample and, whether they were normalised by peptide inhibitors, TF activity analysis was performed using SCENIC v.0.9.6 (Python 3.6). The resulting matrix of cells and TFs was binarised using the recommended threshold calculation in R (v.4.0.3).

### RNA extraction and bulk RNA sequencing

Human fetal cortical neurons (*n* = 1 biological sample due to limited tissue availability) were treated with PBS (control), α-syn monomer, or α-syn PFFs for 14 days prior to RNA extraction. After removing the culture medium, 350 μL of RLT Plus buffer containing β-mercaptoethanol (Qiagen, Cat. No. 74134) was added to each well. Cells were homogenised using a 21G needle, collected, and RNA was purified using the RNeasy Plus Universal Mini Kit (Qiagen, Cat. Nos. 74134, 74136) according to the manufacturer’s instructions. Briefly, samples were mixed with 100 μL gDNA Eliminator Solution and passed through a gDNA Eliminator spin column by centrifugation (2 min at 12,000 × *g*). Lysates were then combined with 70% ethanol (1:1 volume) and transferred to RNeasy spin columns for total RNA purification. RNA concentration and purity were assessed using a NanoDrop spectrophotometer.

Bulk RNA-sequencing was performed at the Jeffrey Cheah Biomedical Centre, Genomics Core Facility, University of Cambridge. Poly-A-selected RNA libraries were prepared and sequenced on an Illumina NovaSeq X platform (SLX_NovaSeqX_1.5B, up to 100 cycles).

### Human postmortem PD dataset

Gene expression data were obtained from postmortem brain tissue provided by the Cambridge Brain Bank, with ethical approval from the London–Bloomsbury Research Ethics Committee (REC reference no. 16/LO/0508). Donors with Lewy body pathology (*n* = 25) had a clinical diagnosis of Parkinson’s disease and were compared to age- and sex-matched control donors without neurological illness (*n* = 18). A neuropathologist confirmed Braak staging, co-existing proteinopathies, and other pathologies. Fresh frozen tissue was dissected from the substantia nigra, putamen, amygdala, and prefrontal cortex (Brodmann area 46). Single-nucleus RNA sequencing was performed using the 10x Genomics Chromium Next GEM platform (3’ kit v3.1, PN-1000268), with ~8500 nuclei loaded per sample. Libraries were prepared with unique dual indices and sequenced on a Novaseq6000 or Novaseq X Plus. A detailed description of dataset generation and processing is available in our preprint^40^.

### Gene set enrichment analysis (GSEA)

Differential gene expression analysis was performed independently on two datasets: fetal human cortical neuron models and postmortem brain tissue samples. Following the generation of differentially expressed genes (DEGs) for each sample set, both DEG lists were analysed using a Gene Set Enrichment Analysis (GSEA) to identify significantly enriched pathways. The analysis was conducted using the standard GSEA software platform, allowing for the systematic comparison of gene expression patterns between the experimental conditions and reference gene sets. This approach enabled the identification of biological pathways and processes that were significantly altered in both the in vitro neuronal models and postmortem samples.

#### REsistance through COntext DRift (RECODR)

RECODR was run on all conditions according to the methodology described in ref. ^22^. Briefly, co-expression graph networks were made using the Python package NetworkX from gene-pairs co-expressed with a Spearman correlation above 0.2 and communities generated from each graph network. Next, Node2Vec was used to run biased random walks from each node with the following parameters, ‘walk_length’ set to 80, ‘num_walks’ set to 400, ‘p’ set to 1 and ‘q’ set to 0.7. Word2vec was trained using Gensim with a context window of 10 and 25 epochs and default remaining parameters. Model alignment was run using the Procrustes method to align word vectors between models.

#### Statistical analysis and reproducibility

Statistical analyses were performed using IBM SPSS Statistics Version 29.0 for macOS and GraphPad Prism Version 9.4.1 for macOS. Statistical tests for each analysis are specified in the corresponding figure legends. One-way ANOVA followed by Tukey’s multiple comparisons test was the primary statistical method employed, with Dunnett’s multiple comparisons test used where appropriate. For all experiments, *n* represents independent biological samples derived from different human subjects, with a minimum of three biological replicates (*n* ≥ 3) per experiment. Statistical significance was determined based on the specific tests employed, with exact *p* values reported in the figure legends. To ensure the conclusions were not driven by single data points, we primarily used parametric tests (e.g., ANOVA) which are robust, and we always visually inspected the effect sizes and checked if the same conclusion held true using non-parametric alternatives where appropriate. Statistical graphs were formatted consistently throughout the manuscript, with representative colours standardised for each experimental group where possible. For data generated from analytical platforms that automatically colour assign data, minor deviations in colour were retained to preserve the original output, but for key figures these colours were manually adjusted for clarity.

### Reporting summary

Further information on research design is available in the Nature Portfolio Reporting Summary linked to this article.

## Supplementary information

Supplementary Information

Description of Additional Supplementary Files

Supplementary Data

Reporting Summary

## Data Availability

Raw single-cell RNA sequencing (scRNA-seq) data generated in this study are deposited in ArrayExpress (EMBL-EBI) under accession number E-MTAB-16863. Differential gene expression lists, including statistical outputs and normalised expression values, are provided in the Supplementary Data (Excel file). All remaining data supporting the findings of this study are contained within the manuscript and its Supplementary Materials.

## Code availability

No custom code was developed for this study. All figures and statistical analyses were generated using IBM SPSS Statistics Version 29.0 and GraphPad Prism Version 9.4.1. scRNA-seq data were analysed using established workflows in Seurat (versions 3.1/4.0.1), following publicly available tutorials.
