# Supplementary material for: Early α-synuclein–mediated mitochondrial dysfunction in a human cell model of Parkinson’s disease dementia
Source: Commun Biol. 2026 May 12;9:1098. doi: 10.1038/s42003-026-10134-x (PMC13470013; doi:10.1038/s42003-026-10134-x)
Supplement: Supplementary file 1 [file 42003_2026_10134_MOESM1_ESM.pdf]

Supplementary Information

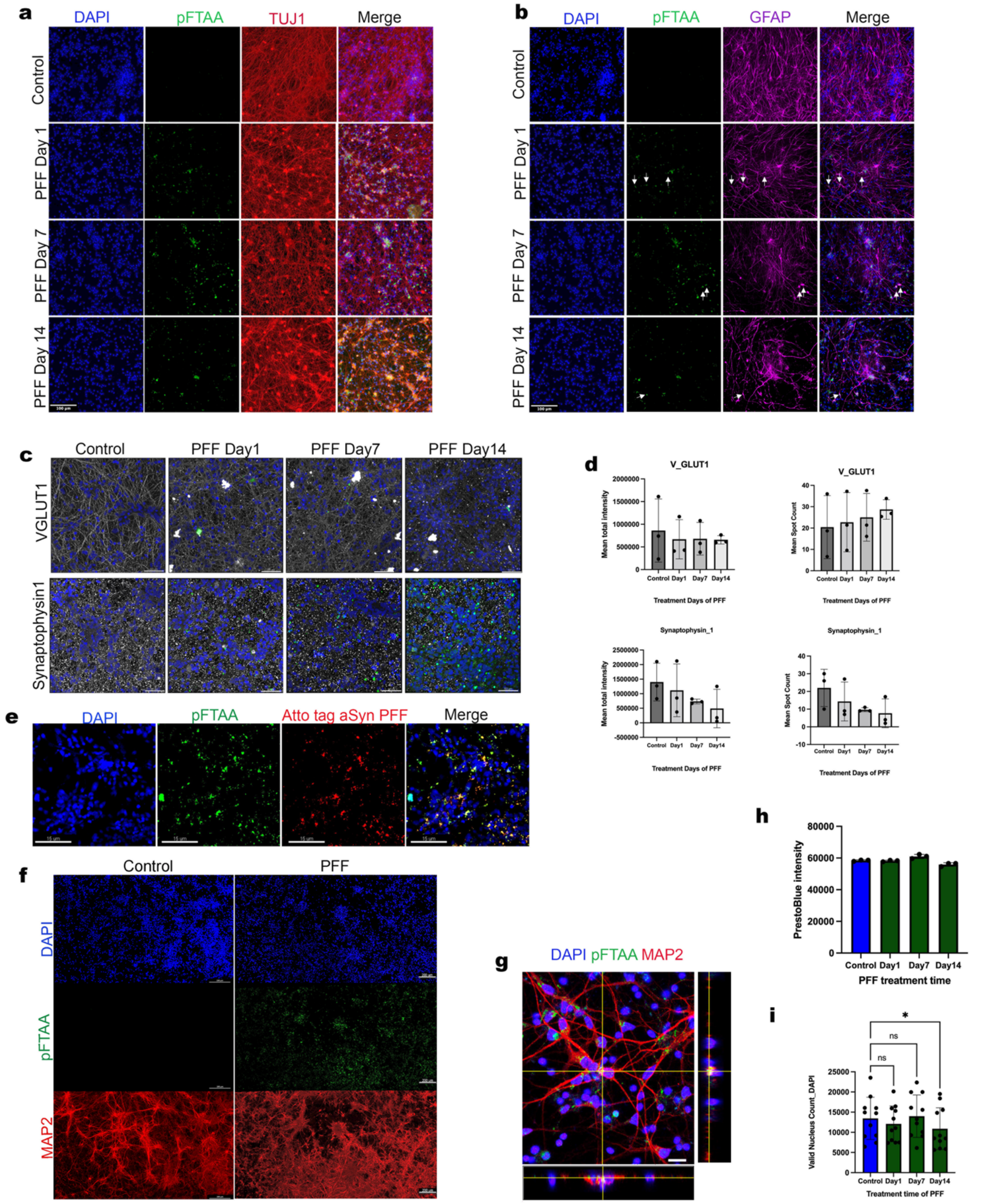

Supplementary Fig.1 | Characterisation of α-synuclein PFF-Seeding in a Fetal Human Model
(a) Representative Cellomics images of fetal human cortical neurons seeded with α-syn PFFs, immunostained with the neuronal marker Tuj1 (red) and the fibril marker pFTAA (green). Quantitative data on Tuj1-pFTAA colocalisation is shown in Figure 1. Nuclei are labelled with DAPI (blue). Scale bar: 100 μm. **(b)** Fetal human cortical cells seeded with α-syn PFFs and immunostained with the

astrocyte marker GFAP (purple). pFTAA (green) PFF-positive, colocalising with GFAP (white arrows). DAPI (blue) labels nuclei. Scale bar: 100 μm. **(c)** Representative confocal images of fetal human cortical neurons treated with α-syn PFFs for 1, 7, and 14 days, immunostained with synaptic markers Synaptophysin-1 (white) and VGLUT1 (white), pFTAA (green), and DAPI (blue). Scale bar: 50 μm. **(d)** Quantification of synaptic markers (VGLUT1 and Synaptophysin-1) based on mean intensity and spot count in fetal human neurons seeded with PFFs for 1, 7, and 14 days. No statistically significant differences were observed among treatment conditions. **(e)** Representative confocal images showing pFTAA-positive fibrils (green) overlapping with Atto-594-tagged α-syn PFFs (red). DAPI (blue) labels nuclei. pFTAA labels both seeded PFF and endogenous α-syn aggregates induced by PFF seeds, while Atto-594 specifically marks exogenous seeds. Neurons were treated with Atto-594-labeled PFFs for 1, 7, and 14 days. Scale bar: 15 μm. **(f)** High-resolution wide-field confocal imaging of fetal human neurons treated with α-syn PFFs for 14 days, showing an overview of the plated cells. Neurons are stained with MAP2 (red), pFTAA (green), and DAPI (blue). A control condition without PFF seeding is also shown. Scale bars: 200 μm (PFF-treated), 100 μm (control). **(g)** Z-stack view demonstrating pFTAA-positive internalised fibrils (green) colocalising with the nuclear marker DAPI (blue) and neuronal marker MAP2 (red). pFTAA signals are observed within the same z-plane as the nucleus. Scale bar: 30 μm. **(h)** Presto Blue (PB) viability assay and cell number quantification in human fetal cortical cells treated with α-syn PFFs over different time points. This assay measures the metabolic activity of the cell (mitochondrial reduction of resazurin). A reduction in the PB signal indicates metabolic compromise and cellular stress, which often precedes definitive cell death. PB signal intensity was measured using a Fluostar Omega microplate fluorescent reader, and showed no significant differences across all conditions. **(i)** Cell number was quantified using Cellomics™ automated analysis software by measuring DAPI-stained nuclei. This assay measures the absolute number of nuclei. DAPI only stains the nuclei of all cells present. A reduction in the DAPI count at a fixed time point is a clear and definitive readout of irreversible cell loss (apoptosis/necrosis). A significant decrease in DAPI count was observed at Day 14 of PFF treatment compared to the control group (Dunnett's multiple comparisons test, *$p = 0.0485$), while no significant differences were detected at other time points.

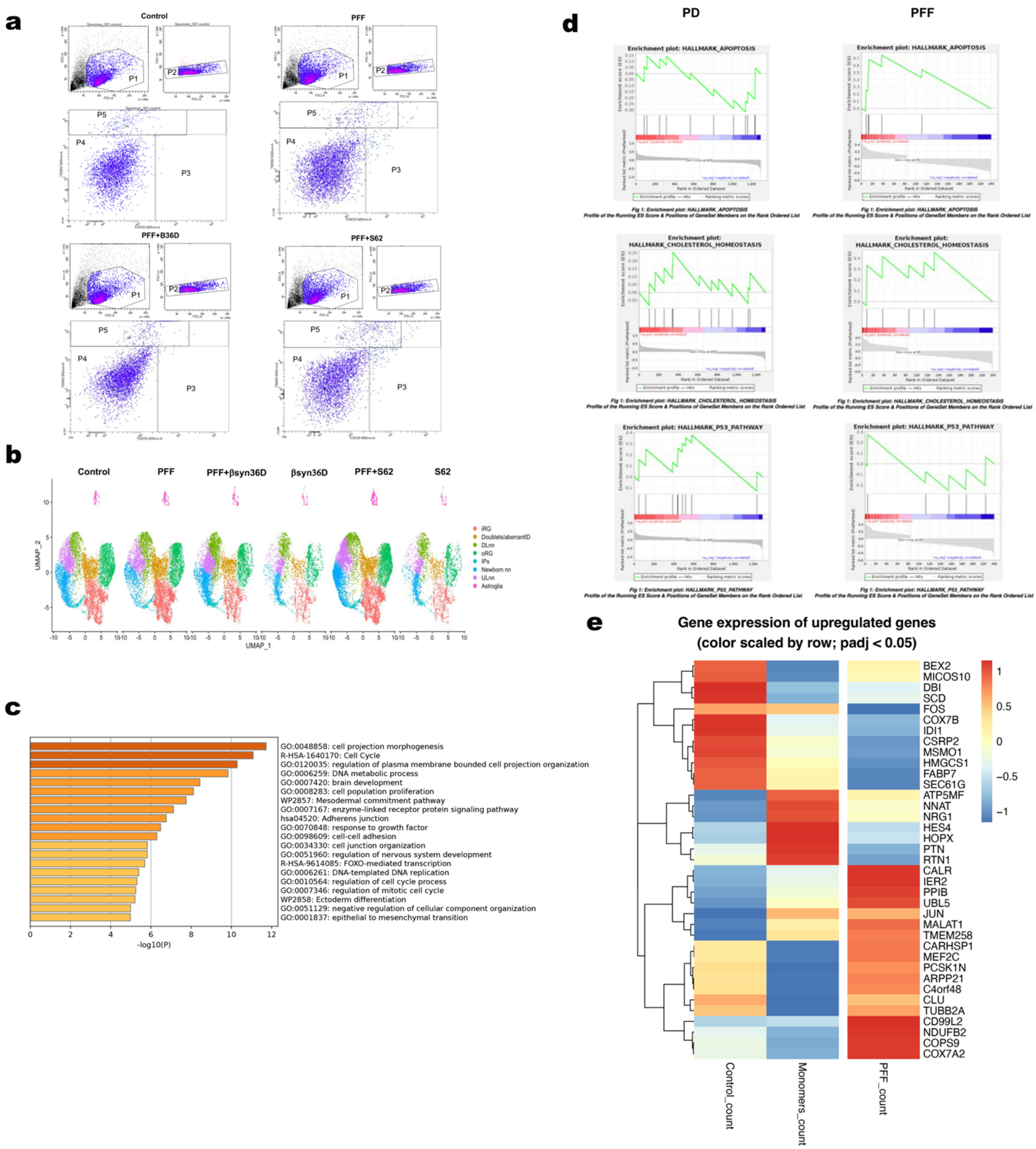

Supplementary Fig.2 | Single-cell characterisation of human fetal cortical cells treated with α-syn PFFs and inhibitors

(a) Flow cytometry (FACS) results assessing sample viability prior to scRNA-seq. Representative gating strategy using FACSDiva 9.0 software for human fetal cortical cells treated with α-syn PFFs and inhibitors. The plots summarise the proportions of different populations: P1 represents cells, excluding smaller debris; P2 represents singlets, excluding doublets; P3 represents pFTAA-positive (α-syn fibril-positive) cells detected using the 488 nm laser; P4 represents live cells, identified by the absence of far-red Zombie dye (640 nm laser); and P5 represents dead cells, stained positively with far-red Zombie dye (640 nm laser). In the control condition, 93.3% of cells were viable, with 0.1% pFTAA-positive cells. In the PFF-seeded condition, 92.4% of cells were viable, with 1.5% pFTAA-

positive cells. The PFF + Bsyn36D inhibitor condition showed 93.4% viability, with 0.3% pFTAA-positive cells. The PFF + S62 inhibitor condition had 87.9% viability, with 2.5% pFTAA-positive cells. **(b)** UMAP clustering analysis illustrates the characterisation of different cell types in human fetal cortical cultures treated with α-syn PFFs, with and without inhibitors. The left panel shows distinct cell populations, including deep-layer neurons (DLnn), upper-layer neurons (ULnn), newborn neurons (Newborn nn), astroglia, intermediate progenitors (IPs), inner radial glia (iRG), and outer radial glia (oRG). The right panel presents UMAP representations of cell type distribution across different treatment conditions: sample 1 (control), sample 2 (PFF), sample 3 (PFF + B36D), sample 4 (B36D), sample 5 (PFF + S62), and sample 6 (S62). B36D (sample 4), and S62 (sample 6) exhibited lower cell counts due to technical issues during the initial sample collection. Each dot represents a single cell, colour-coded by cell type. **(c)** Pathway enrichment analysis identifying significant biological processes associated with the 252 disconnected genes in the PFF-treated condition that altered connectivity compared to the control (Fig. 6c, red dots at -2). **(d)** Gene Set Enrichment Analysis (GSEA) comparing pathways between the PFF-treated model and post-mortem PD samples reveals common but non-significant pathways, including apoptosis, cholesterol homeostasis, and the p53 signalling pathway. **(e)** Heatmap showing the expression of upregulated genes identified in the scRNA-seq dataset (as in Fig. 2c). Bulk RNA-seq analysis confirms that these genes (from *CALR* to *COX7A2*) are upregulated under the α-syn PFF condition but not following monomeric α-syn treatment. Red indicates increased gene expression, demonstrating that these transcriptional changes are driven by α-syn PFFs rather than α-syn monomers.

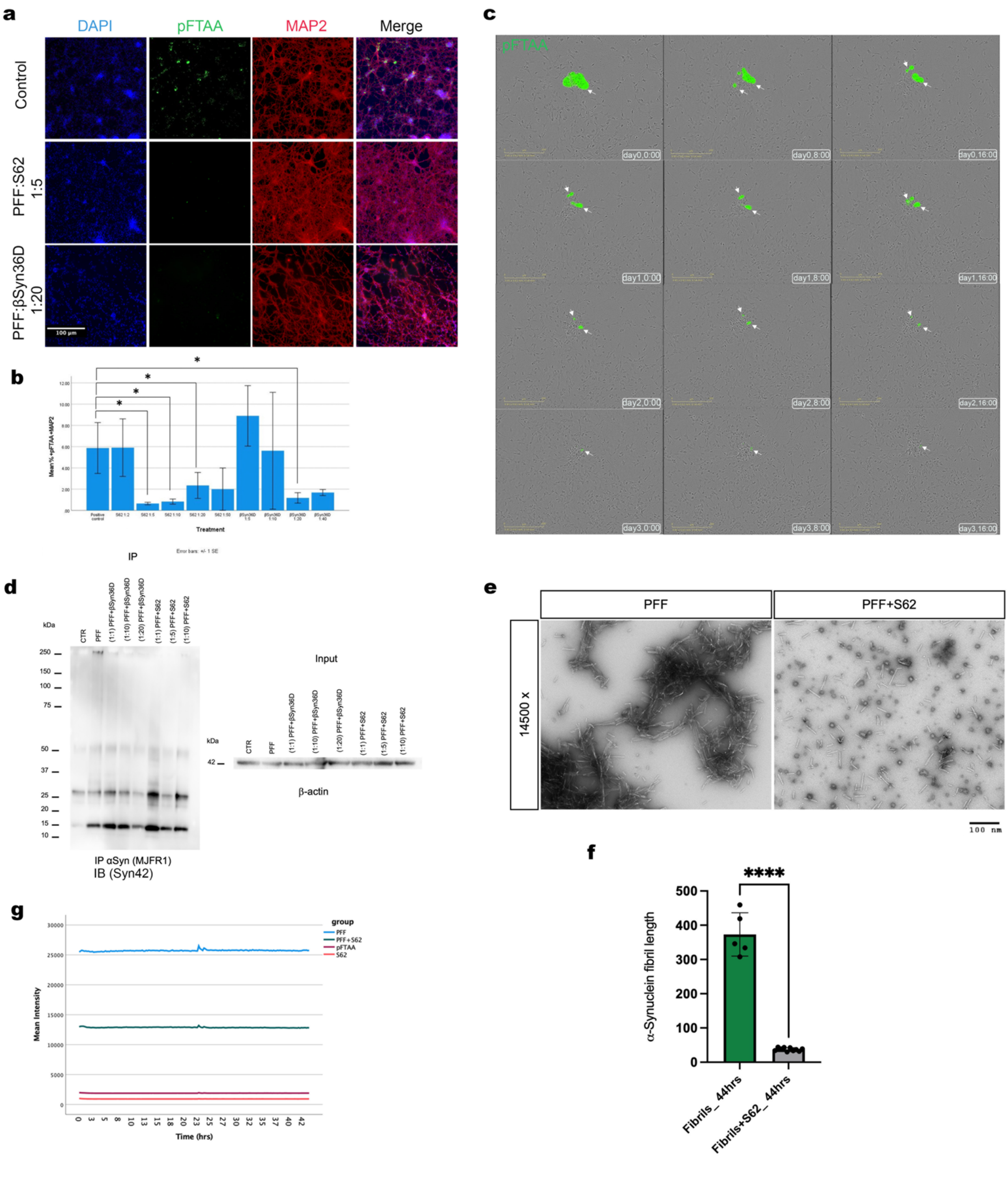

Supplementary Fig.3 | Peptide inhibitor effects on α-synuclein PFF aggregation and disassembly.

(a) Representative immunofluorescence images (20×) of human fetal cortical neurons seeded with α-syn PFFs and treated simultaneously with different concentrations of peptide inhibitors B36D and S62. Fixed cells were immunolabeled with DAPI (nuclear marker, blue), pFTAA (fibril dye, green), and MAP2 (neuronal marker, red). **(b)** Quantification of pFTAA signal intensity in MAP2-positive regions was performed using Cellomics™ software across all experimental conditions. Statistical analysis was conducted using one-way ANOVA and a Dunnett's multiple comparisons test, with statistical significance set at $*p = 0.04$. All statistical comparisons were made against PFF-treated cultures without inhibitors. Scale bar: 100 μm. **(c)** Time-lapse imaging sequence showing B36D-mediated (ratio of 1:20, PFF: B36D) disaggregation of α-syn PFFs (70 nm) in human fetal cortical neurons. Images were captured every 8 hours for the first 3 days post-treatment. PFF aggregates were visualised using pFTAA (green). Scale bar, 200 μm. **(d)** Immunoprecipitation (IP) and Western blot analysis of α-syn aggregates following treatment with different peptide inhibitor ratios. High-molecular-weight (HMW) α-syn aggregates (250 kDa) were detected only in the PFF-treated condition, while no such bands were observed in groups treated with B36D or S62. Monomeric α-syn (15 kDa) was detected in all conditions. β-actin was used as a loading control. Non-specific bands corresponding to the immunoprecipitation antibody heavy (50 kDa) and light (25 kDa) chains were observed. Quantification is presented in Fig.4c. **(e)** Transmission electron microscopy (TEM) images showing α-syn fibrils incubated alone or in the presence of S62. Fibrils treated with S62 exhibited a marked reduction in length. **(f)** Quantification of fibril length from TEM images using ImageJ software. Samples were taken from two experimental conditions: untreated α-syn fibrils and α-syn fibrils incubated with S62 for 44 hours, as illustrated in (d). One-way ANOVA with Tukey's multiple comparisons test revealed a statistically significant reduction in fibril length in the S62-treated condition ($***p < 0.0001$). Scale bar: 100 nm. **(g)** pFTAA kinetic assay assessing the effect of S62 on α-syn fibrils (unsonicated PFFs) in a cell-free system. pFTAA fluorescence intensity was monitored in a microplate reader. The light blue line represents the α-syn fibrils-only positive control, while the green line represents the α-syn fibrils + S62 condition (1:5 ratio). The red and purple lines correspond to the negative controls, S62 alone and pFTAA alone, respectively.

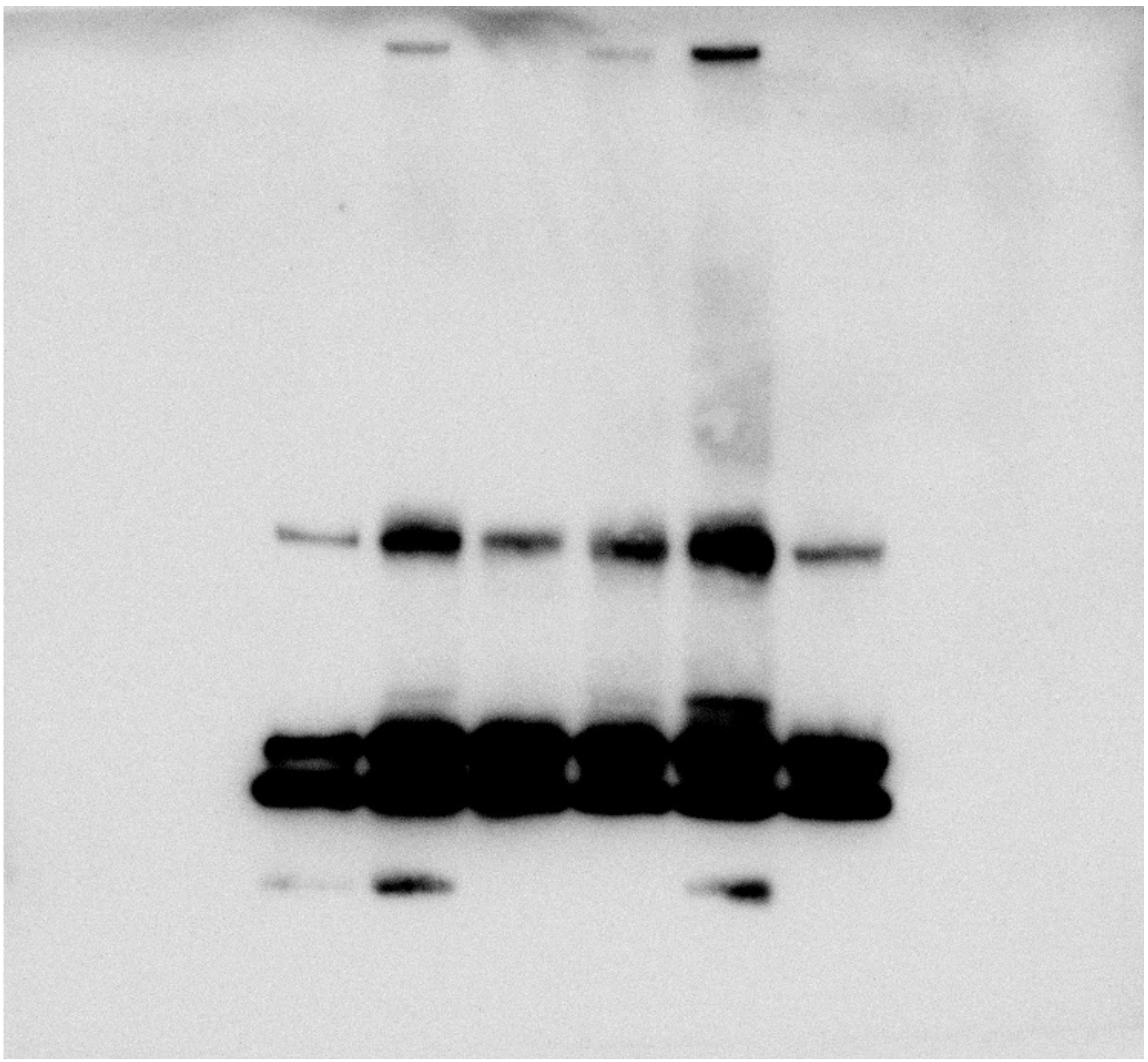

Supplementary Fig.4 | Full-length blots corresponding to Fig. 4b. Top panel: immunoprecipitated (IP) samples; Bottom panel: input samples.

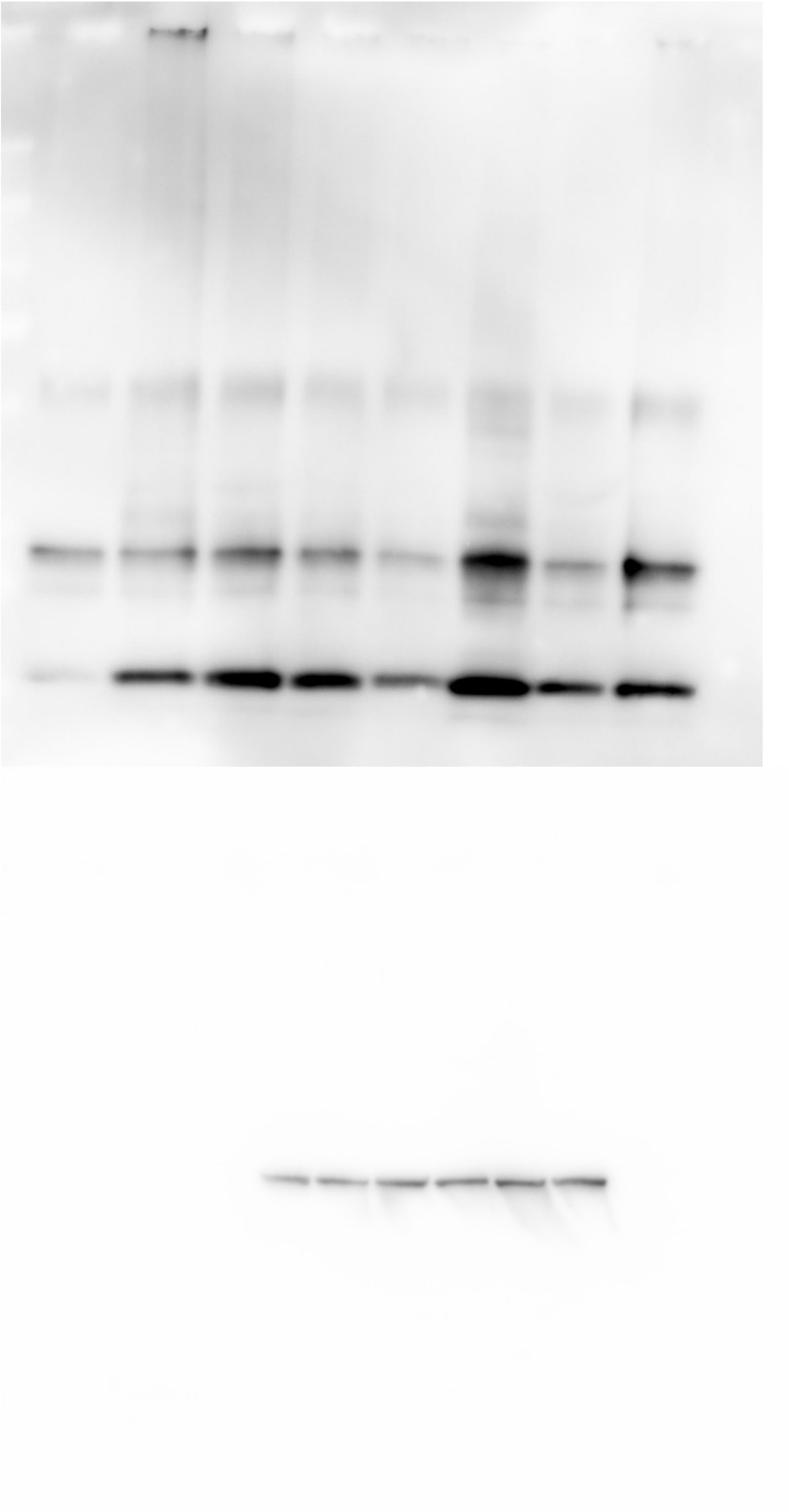

Supplementary Fig. 5 | Full-length blots corresponding to Supplementary Fig. 3d. Top: immunoprecipitated (IP) samples; Bottom: input samples.
