# Supplementary material for: Early α-synuclein–mediated mitochondrial dysfunction in a human cell model of Parkinson’s disease dementia
Source: Commun Biol. 2026 May 12;9:1098. doi: 10.1038/s42003-026-10134-x (PMC13470013; doi:10.1038/s42003-026-10134-x)
Supplement: Supplementary file 3 [file 42003_2026_10134_MOESM2_ESM.docx]

**Description of Additional Supplementary File**

File name: Supplementary Data

Description: Source Data: raw data for all figures
